# Dietary protein restriction throughout intrauterine and postnatal life results in potentially beneficial myocardial tissue remodeling in the adult mouse heart

**DOI:** 10.1038/s41598-019-51654-3

**Published:** 2019-10-22

**Authors:** Maria Hennig, Lea Ewering, Simon Pyschny, Shinya Shimoyama, Maja Olecka, Dominik Ewald, Manuela Magarin, Anselm Uebing, Ludwig Thierfelder, Christian Jux, Jörg-Detlef Drenckhahn

**Affiliations:** 1 Department of Pediatric Cardiology, University Hospital Münster, Münster, Germany; 2 Max-Delbrück-Center for Molecular Medicine, Berlin, Germany; 3 Department of Pediatric Cardiology, Justus Liebig University, Gießen, Germany; 4 Department of Pediatric Cardiology, Gunma Children’s Medical Center, Gunma, Japan; 5 Department of Pediatrics, Gunma University Graduate School of Medicine, Gunma, Japan

**Keywords:** Intrauterine growth, Heart development, Cardiac hypertrophy

## Abstract

Diet composition impacts metabolic and cardiovascular health with high caloric diets contributing to obesity related disorders. Dietary interventions such as caloric restriction exert beneficial effects in the cardiovascular system, but alteration of which specific nutrient is responsible is less clear. This study investigates the effects of a low protein diet (LPD) on morphology, tissue composition and function of the neonatal and adult mouse heart. Mice were subjected to LPD (8.8% protein) or standard protein diet (SPD, 22% protein) throughout intrauterine and postnatal life. At birth LPD female but not male offspring exhibit reduced body weight whereas heart weight was unchanged in both sexes. Cardiomyocyte cross sectional area was increased in newborn LPD females compared to SPD, whereas proliferation, cellular tissue composition and vascularization were unaffected. Adult female mice on LPD exhibit reduced body weight but normal heart weight compared to SPD controls. Echocardiography revealed normal left ventricular contractility in LPD animals. Histology showed reduced interstitial fibrosis, lower cardiomyocyte volume and elevated numbers of cardiomyocyte and non-myocyte nuclei per tissue area in adult LPD versus SPD myocardium. Furthermore, capillary density was increased in LPD hearts. In conclusion, pre- and postnatal dietary protein restriction in mice causes a potentially beneficial myocardial remodeling.

## Introduction

Diet composition has a major impact on metabolic and cardiovascular health in humans and influences the pathogenesis of various chronic diseases including diabetes mellitus, hypertension and coronary heart disease^1^. One of the most obvious examples is obesity induced by high caloric diets (containing high levels of saturated fats and refined carbohydrates, i.e. sugar), which causes metabolic syndrome and is associated with most of the diseases listed above^2^. Furthermore, a high fat diet (HFD) has been shown to execute negative effects in animal models by increasing body weight and fat deposition, increasing blood glucose and lipid levels, causing insulin resistance and elevating blood pressure^3,4^. In the rodent heart, HFD causes variable outcomes likely caused by differences in diet composition, onset and duration as well as species, gender or genetic background^3^. Nevertheless, HFD appears to promote cardiac hypertrophy resulting in impaired contractility under baseline conditions^5,6^ and increase myocardial susceptibility to ischemia^7,8^.

Caloric restriction (CR) has been shown to improve metabolic and cardiovascular health in humans^9,10^ and extend lifespan in various model organisms^11^. In rodents, for example, CR prevents hypertension and cardiac hypertrophy in spontaneously hypertensive rats^12^, improves heart function after ischemia/reperfusion injury or myocardial infarction^13,14^ and ameliorates ageing associated cardiac dysfunction^15,16^. Furthermore, intermittent fasting has been shown to exert cardioprotective effects in the rat heart^17,18^. Interestingly, a Mediterranean diet is associated with beneficial influences on human cardiovascular health and lifespan^19^. Although a Mediterranean diet might contain less calories compared to most western diets, it has become increasingly clear that its composition (high amounts of whole grains, vegetables, fruits, nuts and olive oil; moderate amounts of seafood, fish, poultry and dairy products; little red meat and sweets) likely mediates the positive impact^19^.

In order to identify specific diet components that mediate the beneficial effects of CR, carbohydrate and protein content has been modified. Interestingly, low carbohydrate/high protein diets appear to increase the risk of cardiovascular disease in humans^20,21^. In contrast, dietary protein restriction has been associated with longevity in various model organisms as well as humans by reducing overall mortality and cancer incidence^22–24^. It even appears that specific amino acids, such as branched-chain amino acids or methionine, might be primarily responsible for the positive outcome of dietary protein restriction^23^.

Although beneficial influences of a low protein diet (LPD) on overall metabolic health and longevity have been proposed in animal models and humans, the specific effects on the heart have not been investigated. The aim of this study was to expose mice to dietary protein restriction both throughout intrauterine development as well as postnatal life and investigate the consequences for the heart at birth and in early adulthood. Our data revealed normal left ventricular function and various potentially beneficial alterations in cell composition and vascularization of the LV myocardium in adult mice constantly on LPD.

## Results

### Reduced protein content in the diet is not compensated for by increased food intake in female mice

We subjected female mice to LPD (8.8% protein compared to 22% in the standard protein diet (SPD)) for ≥2 weeks prior to mating and throughout pregnancy. Mice could potentially compensate the effect of the altered diet composition by increasing food consumption, such that protein uptake would not be different compared to the SPD group. We measured food intake in pregnant and non-pregnant adult female mice but did not find a difference between groups (Supplementary Fig. S1a and S1b). Consequently, the calculated daily protein uptake per mouse normalized to body weight was 44.7 ± 3.2 mg/g BW in SPD versus 14.7 ± 0.7 mg/g BW in LPD non-pregnant females and 29.2 ± 0.6 mg/g BW versus 10.4 ± 1.2 mg/g BW in pregnant females, respectively.

### LPD reduces body weight in female but not male offspring at birth and does not affect heart size or cardiac morphology

**Figure 1:**
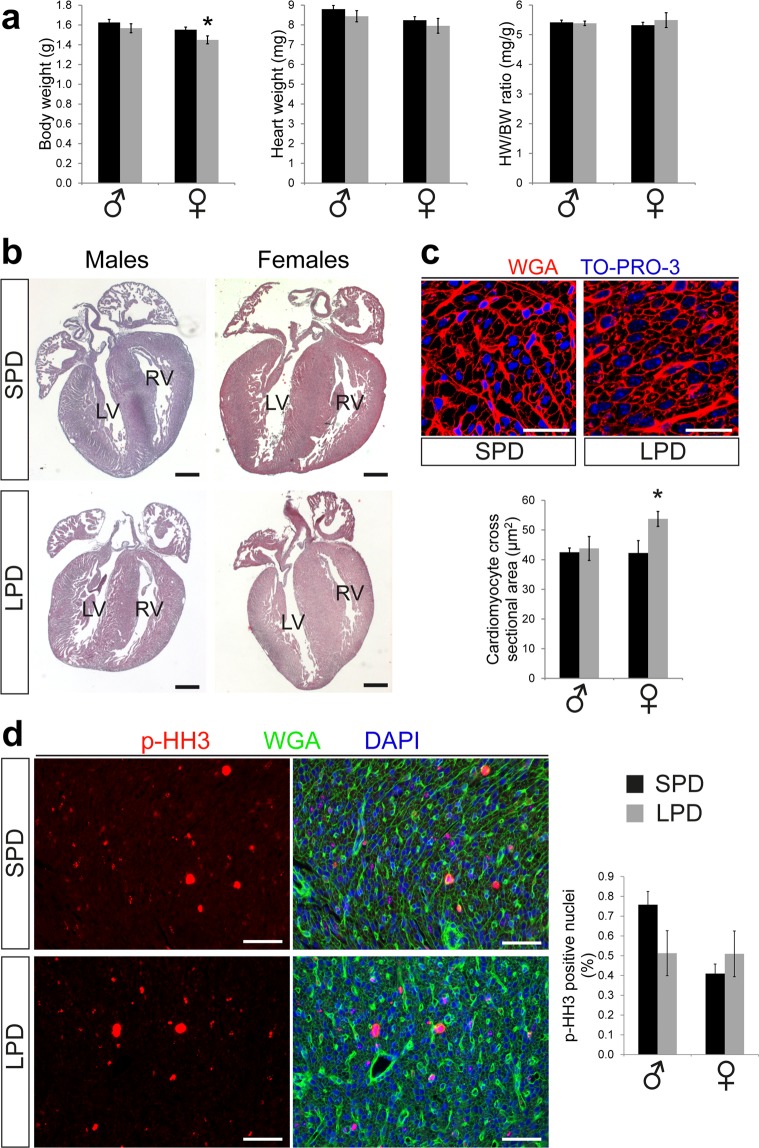
Normal cardiac size and morphology but increased cardiomyocyte cross sectional area in LPD compared to SPD neonatal female mice. (**a**) Body and heart weight were determined in neonatal mice from SPD and LPD pregnancies on postnatal day 1. Body weight was reduced in LPD compared to SPD females but not males. Heart weight (absolute as well as normalized to body weight) is not affected by LPD (SPD ♂ n = 22, LPD ♂ n = 17, SPD ♀ n = 20, LPD ♀ n = 11 litters, *P < 0.05 versus SPD females). (**b**) Representative overview images of male and female P1 hearts on SPD and LPD (H&E staining, RV = right ventricle, LV = left ventricle, scale bar = 500 µm). (**c**) Fluores**c**ence images of cross-sectioned cardiomyocyte in the LV myocardium of neonatal SPD and LPD female hearts. Cell membranes are stained in red using WGA and nuclei in blue using TO-PRO-3. Cardiomyocyte cross sectional area is increased in female but not male hearts on LPD compared to SPD (SPD ♂ n = 5, LPD ♂ n = 6, SPD ♀ n = 6, LPD ♀ n = 6 litters, scale bar = 25 µm, *P < 0.05 versus SPD females). (**d**) Immunofluorescence staining for phosphorylated histone H3 (p-HH3, red) was used to detect cell proliferation in the LV myocardium of neonatal SPD and LPD mice. Cell membranes were stained in green and nuclei in blue using WGA and DAPI, respectively. No difference in proliferation rates was observed between groups (SPD ♂ n = 5, LPD ♂ n = 6, SPD ♀ n = 6, LPD ♀ n = 5 litters, scale bar = 50 µm).

To explore the consequences of intrauterine protein restriction on the neonatal heart, we analyzed mice from SPD and LPD litters on postnatal day 1 (P1). Importantly, litter size was comparable between both groups (Supplementary Fig. S1c). Although maternal LPD during pregnancy has been shown to reduce birth weight in both sexes^25^, we only observed a reduction in body weight in female but not male offspring (Fig. 1a). Heart weight as well as heart weight to body weight ratios were not different between SPD and LPD hearts in both sexes at P1 (Fig. 1a). Histological examinations confirmed normal cardiac morphology, left ventricular (LV) dimensions and wall thickness in LPD compared to SPD neonates (Fig. 1b).

### Sex dependent alterations in cardiomyocyte size but not proliferation in newborn LPD compared to SPD mice

Neonatal heart sections were stained with fluorochrome-conjugated wheat germ agglutinin (WGA) to visualize cell membranes and measure cardiomyocyte cross sectional area (CSA) in the LV myocardium. Whereas no difference in cardiomyocyte CSA was observed between SPD and LPD males, CSA was significantly increased in LPD compared to SPD females at P1 (Fig. 1c). The latter indicates a sex specific effect of prenatal LPD on cardiomyocyte size at birth. Proliferation rates in the neonatal heart were investigated using immunostaining for phosphorylated histone H3 (p-HH3) to detect mitotic cells. When normalizing the number of p-HH3 positive nuclei to the total number of nuclei in the left ventricular myocardium (irrespective of cell type), no differences in overall proliferation rates were observed in LPD compared to SPD male and female P1 hearts (Fig. 1d). In summary, these data indicate that under the experimental conditions used in this study maternal LPD during pregnancy has sex specific effects on cardiomyocyte size but not cell cycle activity in the neonatal heart.

### Normal cell composition in LPD versus SPD myocardium at birth

**Figure 2:**
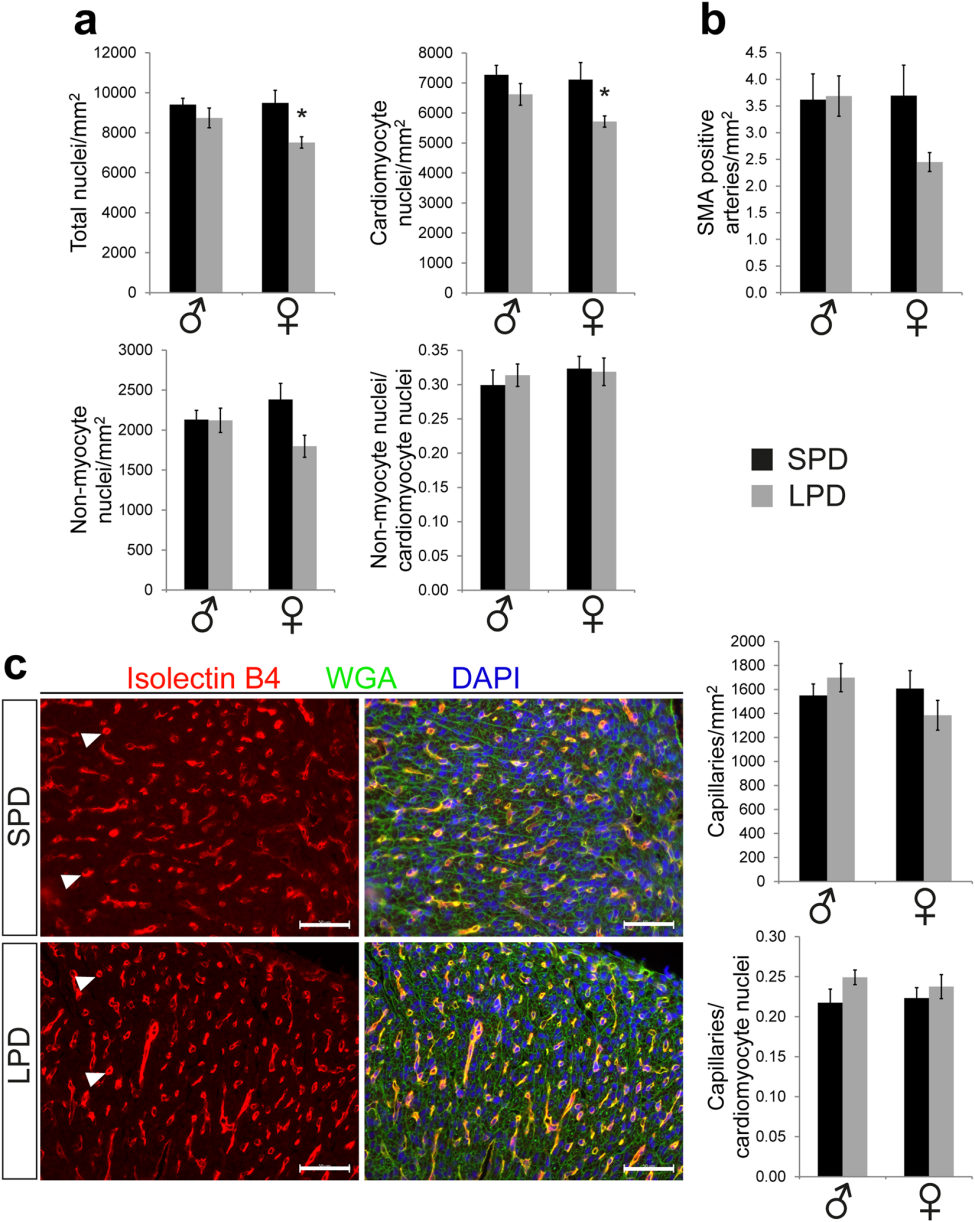
Cellular tissue composition and vascularization in the neonatal LPD heart. (**a**) The total number of nuclei as well as the number of cardiomyocyte nuclei per LV tissue area is reduced in LPD compared to SPD females whereas no difference between diets was observed in males. The number of non-myocyte nuclei per area as well as the ratio of non-myocyte to cardiomyocyte nuclei are not different between groups (SPD ♂ n = 5, LPD ♂ n = 6, SPD ♀ n = 6, LPD ♀ n = 5 litters, *P < 0.05 versus SPD females). (**b**) The num**b**er of smooth muscle actin (SMA) positive arteries within the LV myocardium was not different between SPD and LPD neonates of both sexes (SPD ♂ n = 5, LPD ♂ n = 6, SPD ♀ n = 6, LPD ♀ n = 6 litters). (**c**) Capillarization of the LV myocardium in neonatal SPD and LPD hearts was determined by Isolectin B4 fluorescence staining (red, arrowheads indicate capillary profiles) whereas cell membranes were stained in green using WGA and nuclei in blue using DAPI. No differences in capillary density or the ratio of capillaries per cardiomyocyte nuclei were observed between groups (SPD ♂ n = 5, LPD ♂ n = 6, SPD ♀ n = 6, LPD ♀ n = 5 litters, scale bar = 50 µm).

Cellular tissue composition was determined in the left ventricular myocardium of newborn SPD and LPD hearts. Non-myocytes and cardiomyocytes were differentiated based on WGA staining of cell membranes in combination with DAPI staining of nuclei (see methods and Supplementary Fig. S2). Consistent with increased cardiomyocyte CSA, the number of total nuclei as well as cardiomyocyte nuclei normalized to myocardial tissue area was reduced in LPD compared to SPD female hearts (Fig. 2a). The number of non-myocyte nuclei per tissue area and the ratio of non-myocyte per cardiomyocyte nuclei were not affected by LPD, however (Fig. 2a). Nuclei numbers per area as well as non-myocyte to cardiomyocyte nuclei ratio was not different in LPD versus SPD male myocardium (Fig. 2a), consistent with normal cardiomyocyte size in LPD male hearts. These data indicate appropriate cellular tissue composition in LPD compared to SPD neonatal myocardium, despite alterations caused by increased cardiomyocyte size in LPD females.

### Normal vascularization in newborn LPD compared to SPD myocardium

To evaluate whether prenatal LPD has consequences for myocardial vascularization in the neonatal heart, we investigated the number of coronary arteries in SPD and LPD LV myocardium at P1 based on immunofluorescence staining of smooth muscle cells in vessel walls (Supplementary Fig. S3a). These data revealed a normal number of intramyocardial arteries per tissue area in LPD compared to SPD males and females, although LPD females tended to have fewer arteries compared SPD females (Fig. 2b). Morphology and size of arteries was not different in LPD versus SPD female hearts, however (Supplementary Fig. S3b and S3c). Capillary density was determined based on Isolectin B4 fluorescence staining to detect endothelial cells (Fig. 2c). The number of capillaries normalized to myocardial tissue area as well as the ratio of capillaries per cardiomyocyte nuclei (determined by WGA/DAPI staining as described above) was not different between dietary groups in both sexes (Fig. 2c), indicating normal development of the myocardial microvasculature in LPD hearts. In summary, these data show that prenatal dietary protein restriction does not affect vascularization in the neonatal mouse myocardium.

### Reduced body weight but normal cardiac morphology and function in adult females subjected to pre- and postnatal LPD

Given the sex specific alterations in body weight and cardiomyocyte size observed in LPD females but not males at birth, we subjected female mice born from LPD pregnancies to postnatal LPD until early adulthood (i.e. 11 weeks of age). Another rational to focus on females was the aim to investigate the effect of dietary protein restriction on postnatal compensatory growth of the heart after neonatal cardiac hypoplasia. Therefore, we used mice carrying a heart conditional knockout of the X-linked Holocytochrome c synthase (*Hccs*) gene^26^. Whereas the hemizygous inactivation of *Hccs* in the heart of male mice results in embryonic lethality, heterozygous *Hccs* knockout females (*cHccs*^+/−^) are born with reduced heart size due to a reduced number of cardiomyocytes^27^. Heart size normalizes during postnatal development until early adulthood mainly by compensatory hypertrophy of existing cardiomyocytes^27^. To test whether this process is dependent on dietary protein uptake, we included *cHccs*^+/−^ female mice in this study and subjected them to pre- and postnatal dietary protein restriction.

**Figure 3:**
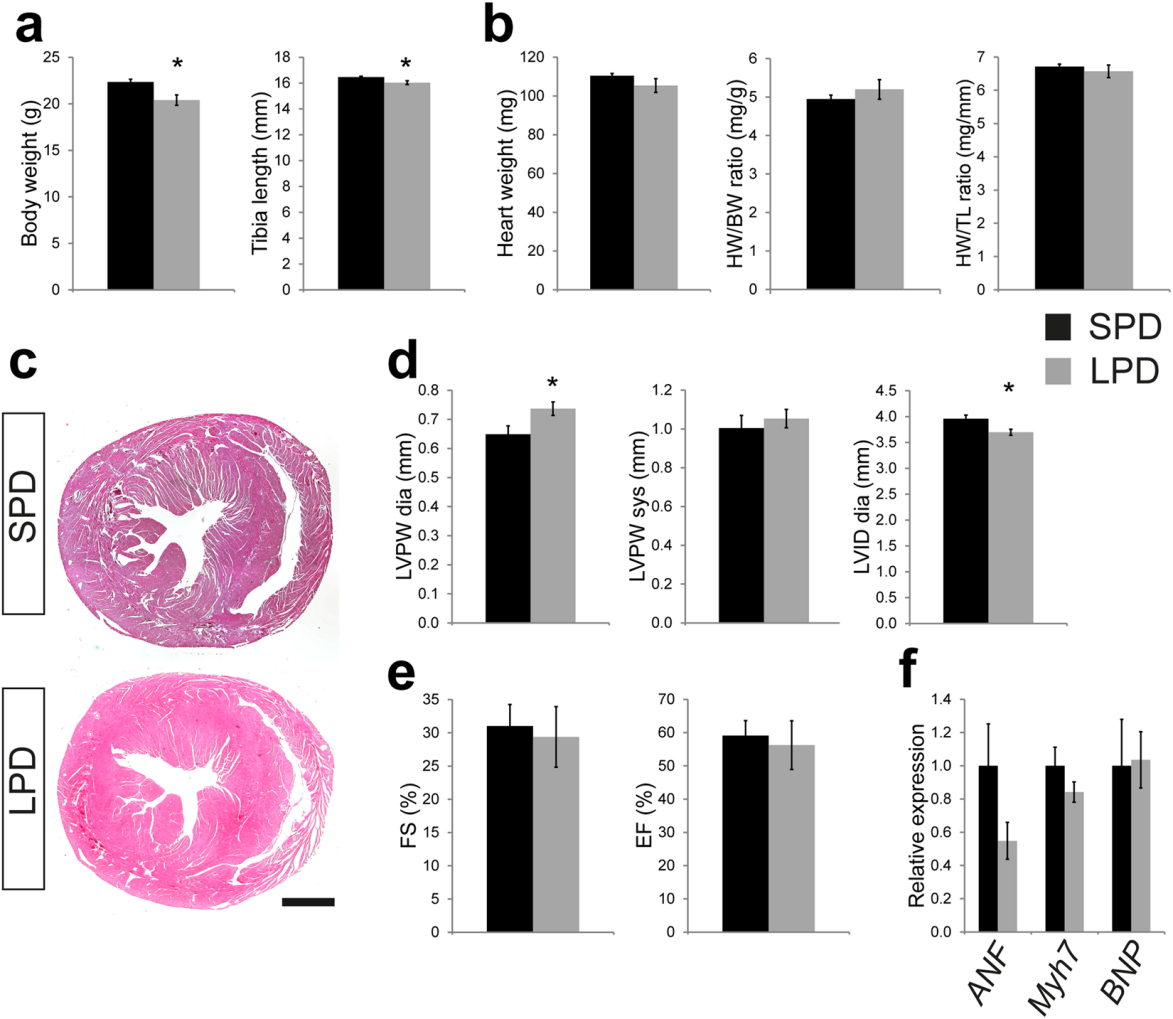
Reduced body weight but normal heart weight and contractility in adult female mice on LPD. (**a**) Body weight and tibia length are reduced in 11 week old LPD compared to SPD female mice (*P < 0.05). (**b**) Absolute heart weight as well as heart weight normalized to body weight (HW/BW) or tibia length (HW/TL) is not different between SPD and LPD females (n = 5 litters per group in (a) and (b)). (**c**) Histologi**c**al analyses revealed no major alterations of gross myocardial morphology in adult LPD compared to SPD mice (H&E staining, scale bar = 1 mm). (**d**) Echocar**d**iographic measurements show a significantly increased left ventricular posterior wall (LVPW) thickness in diastole but not systole as well as a reduced left ventricular internal diameter (LVID) in 11 week old LPD compared to SPD females (*P < 0.05). (**e**) Left ventricular contractility determined by echocardiography is not different between SPD and LPD hearts (FS = fractional shortening, EF = ejection fraction, SPD n = 7, LPD n = 6 mice in (d) and (e)). (**f**) RNA expression of **f**etal genes reactivated in the adult heart upon pathological conditions was not different between adult LPD and SPD hearts (ANF = atrial natriuretic factor, encoded by the *Nppa* gene; BNP = brain natriuretic protein, encoded by the *Nppb* gene; *Myh7* encoding β-myosin heavy chain, SPD n = 4, LPD n = 5 litters).

Pre- and postnatal LPD results in reduced body weight and tibia length (Fig. 3a) but normal absolute heart weight as well as normal heart weight to body weight and heart weight to tibia length ratios in adult wildtype LPD compared to SPD female mice (Fig. 3b). Interestingly, none of these parameters was different in LPD compared to SPD *cHccs*^+/−^ females or in LPD *cHccs*^+/−^ compared to LPD wildtype females (Supplementary Fig. S4). Gross cardiac morphology was not affected by LPD in wildtype mice, as revealed by histological analyses (Fig. 3c). To gain further insights into LV morphology and function, we performed echocardiography in adult LPD and SPD female mice. These data revealed a slight but significant increase in LV wall thickness in diastole but not in systole as well as a slightly reduced LV internal diameter in LPD compared to SPD females (Fig. 3d). Left ventricular contractility, however, was not different between dietary groups (Fig. 3e). Consistently, RNA expression of fetal genes reactivated in the adult heart upon pathological conditions is not different between LPD and SPD females (Fig. 3f). Interestingly, echocardiography furthermore revealed that LV wall thickness, diameter and contractility in *cHccs*^+/−^ female mice were not negatively affected by pre- and postnatal LPD (Supplementary Table S1). The latter suggests that the demands for postnatal compensatory growth and maintenance of normal cardiac function after neonatal cardiac hypoplasia are not negatively influenced by LPD. In summary, pre- and postnatal dietary protein restriction is well tolerated in the murine heart until early adulthood and does not negatively impact on left ventricular function.

### Reduced cardiomyocyte size but normal cardiomyocyte number in adult hearts after pre- and postnatal LPD

**Figure 4:**
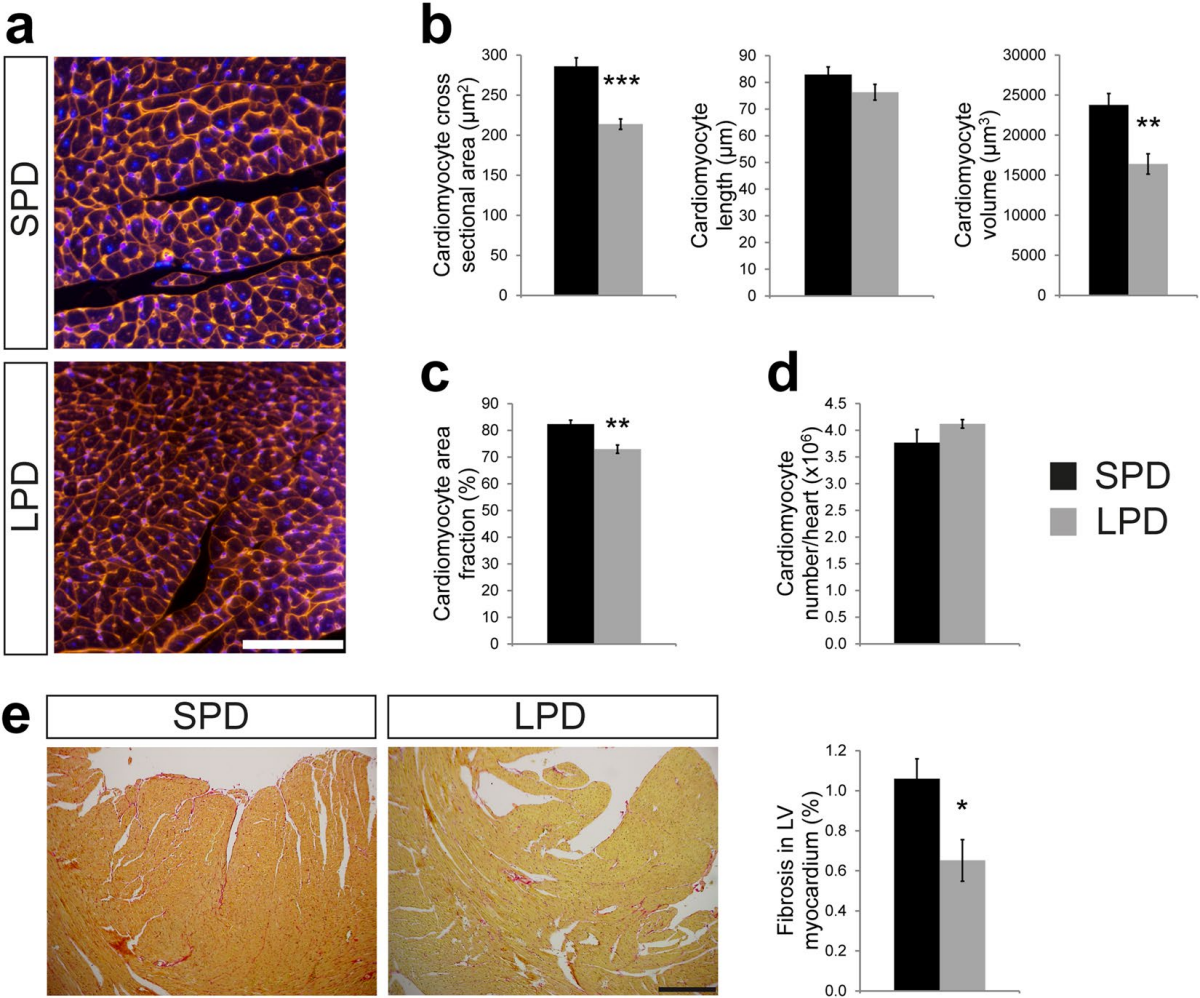
Reduced cardiomyocyte volume but normal number and absence of fibrosis in adult hearts on LPD. (**a**) Representative images of cross-sectioned cardiomyocytes in the LV myocardium of adult SPD and LPD hearts. Cell membranes are stained in red using WGA and nuclei in blue using DAPI (scale bar = 100 µm). (**b**) Cardiomyocyte cross sectional area (SPD n = 6, LPD n = 4 mice) is reduced in adult LPD compared to SPD LV myocardium, whereas cardiomyocyte length (n = 6 mice per group) is unaltered. This results in a significant reduction in calculated cardiomyocyte volume (SPD n = 6, LPD n = 4 mice) in LPD hearts (**P < 0.01, ***P < 0.001). (**c**) Cardiomyocyte area fraction is reduced in tissue sections of LV myocardium in LPD compared to SPD hearts (SPD n = 6, LPD n = 4 mice, **P < 0.01). (**d**) Cardiomyocyte number per heart was calculated based on cardiomyocyte volume, area fraction and heart weight (see methods). No difference was observed between adult LPD and SPD hearts (SPD n = 6, LPD n = 4 mice). (**e**) Representative images of LV myocardium stained with Sirius Red to visualize extracellular matrix deposition (scale bar = 300 µm). Interstitial fibrosis was reduced in LPD compared to SPD hearts (SPD n = 4, LPD n = 6 mice, *P < 0.05).

The consequences of pre- and postnatal LPD on myocardial tissue composition were determined in heart sections of 11 week old female mice. Cardiomyocyte CSA was measured based on WGA staining (Fig. 4a), which revealed a reduced CSA in LPD compared to SPD hearts (Fig. 4b). Cardiomyocyte length was not different between dietary groups (Fig. 4b), as determined by N-cadherin immunofluorescence staining of intercalated discs in longitudinally oriented cardiomyocytes (Supplementary Fig. S5). Consequently, the calculated cardiomyocyte volume was significantly reduced in LPD compared to SPD hearts (Fig. 4b). Furthermore, the cardiomyocyte area fraction was reduced in LPD versus SPD hearts (Fig. 4c), as determined by WGA staining in tissue sections. Based on heart weight, myocardial tissue density, cardiomyocyte volume and cardiomyocyte area fraction, the number of cardiomyocytes per heart was calculated (see methods). This revealed a normal cardiomyocyte number in LPD compared to SPD female hearts (Fig. 4d). Considering reduced cardiomyocyte volume but normal cardiomyocyte number in adult LPD compared to SPD females, this raises the question how normal heart weight and morphology (Fig. 3) is achieved upon LPD conditions. One possible explanation would be the compensatory deposition of extracellular matrix (ECM), which could also explain the reduced cardiomyocyte area fraction. However, quantification of Sirius Red stained tissue sections actually revealed a reduction in myocardial fibrosis in adult LPD versus SPD hearts (Fig. 4e). The latter was confirmed by unaltered RNA and protein expression of various ECM components (i.e. collagen isoforms, fibronectin, osteopontin) and key regulators of fibrosis (TGF-β) in LPD hearts (Supplementary Fig. S6). In summary, pre- and postnatal LPD results in normal cardiomyocyte number but reduced size, which is not compensated by excessive ECM deposition to maintain normal organ size.

### Increased cell density per tissue area in the adult heart after pre- and postnatal LPD

**Figure 5:**
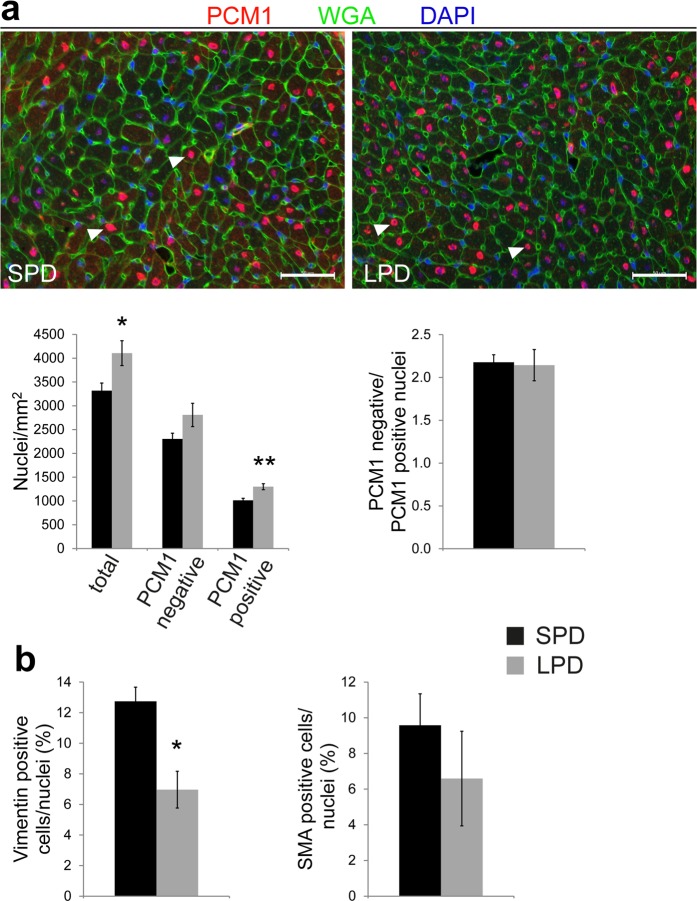
Altered cellular tissue composition in the LV myocardium of adult mice on LPD. (**a**) The number of cardiomyocyte and non-myocyte nuclei per tissue area was determined in LV myocardium of adult SPD and LPD mice based on immunofluorescence staining for PCM1 (red). WGA and DAPI staining was used to label cell membranes (green) and nuclei (blue), respectively. The total number of nuclei as well as the number of PCM1 positive cardiomyocyte nuclei per area was increased in LPD compared to SPD hearts. The number of non-myocyte nuclei per area as well as the ratio of non-myocyte/cardiomyocyte nuclei were not different between groups (SPD n = 3, LPD n = 5 litters, *P < 0.05, **P < 0.01). (**b**) Fibroblasts and myofibroblasts were quantified in the LV myocardium of adult LPD and SPD hearts based on immunofluorescence staining for vimentin and smooth muscle actin (SMA), respectively. The number of vimentin positive cells normalized to the total number of nuclei was reduced in LPD compared to SPD hearts, whereas SMA positive cell numbers were unchanged (n = 4 mice per group, *P < 0.05).

Given normal cardiomyocyte number but reduced cardiomyocyte volume we hypothesized that a non-myocyte cell population might account for normal heart size in adult LPD females. This would be consistent with a reduced cardiomyocyte area fraction in the absence of myocardial fibrosis, as shown above (Fig. 4). Cardiomyocyte and non-myocyte nuclei were differentiated based on WGA and DAPI staining, as described for neonatal hearts (see methods and Supplementary Fig. S7). The number of both non-myocyte nuclei as well as cardiomyocyte nuclei per tissue area was significantly increased in LPD compared to SPD hearts, resulting in a normal non-myocyte/cardiomyocyte nuclei ratio (Supplementary Fig. S7). Given that differentiating myocardial cell types based on WGA and DAPI staining might be inaccurate we verified these data by specifically detecting cardiomyocyte nuclei using immunofluorescence staining for PCM1 (pericentriolar material 1) (Fig. 5a). The latter has been shown to reliably label cardiomyocyte nuclei in the adult heart^28^. This approach confirmed the results based on WGA/DAPI staining (Supplementary Fig. S7), showing increased numbers of total nuclei as well as PCM1 positive cardiomyocyte nuclei per LV tissue area in LPD compared to SPD hearts but normal ratios of non-myocyte (PCM1 negative) nuclei per cardiomyocyte (PCM1 positive) nuclei (Fig. 5a).

Given the increased density of non-myocyte nuclei per tissue area in LPD compared to SPD myocardium, we aimed to identify the underlying cell type. Fibroblasts represent an obvious candidate, however, quantification of vimentin positive fibroblasts based on immunostaining in tissue sections (Supplementary Fig. S8a) revealed a reduced contribution to the myocardial cell populations in LPD compared to SPD females (Fig. 5b). Smooth muscle actin (SMA) positive myofibroblasts (Supplementary Fig. S8b) were unchanged between dietary groups (Fig. 5b). Consistently, RNA and/or protein expression of vimentin and SMA was not different between LPD and SPD adult hearts (Supplementary Fig. S6). In conclusion, pre- and postnatal LPD conditions alter myocardial tissue composition by increasing cardiomyocyte and non-myocyte density, which appears not to involve fibroblasts.

### Increased capillary density per area but normal capillary per cardiomyocyte ratio in adult hearts after pre- and postnatal LPD

**Figure 6:**
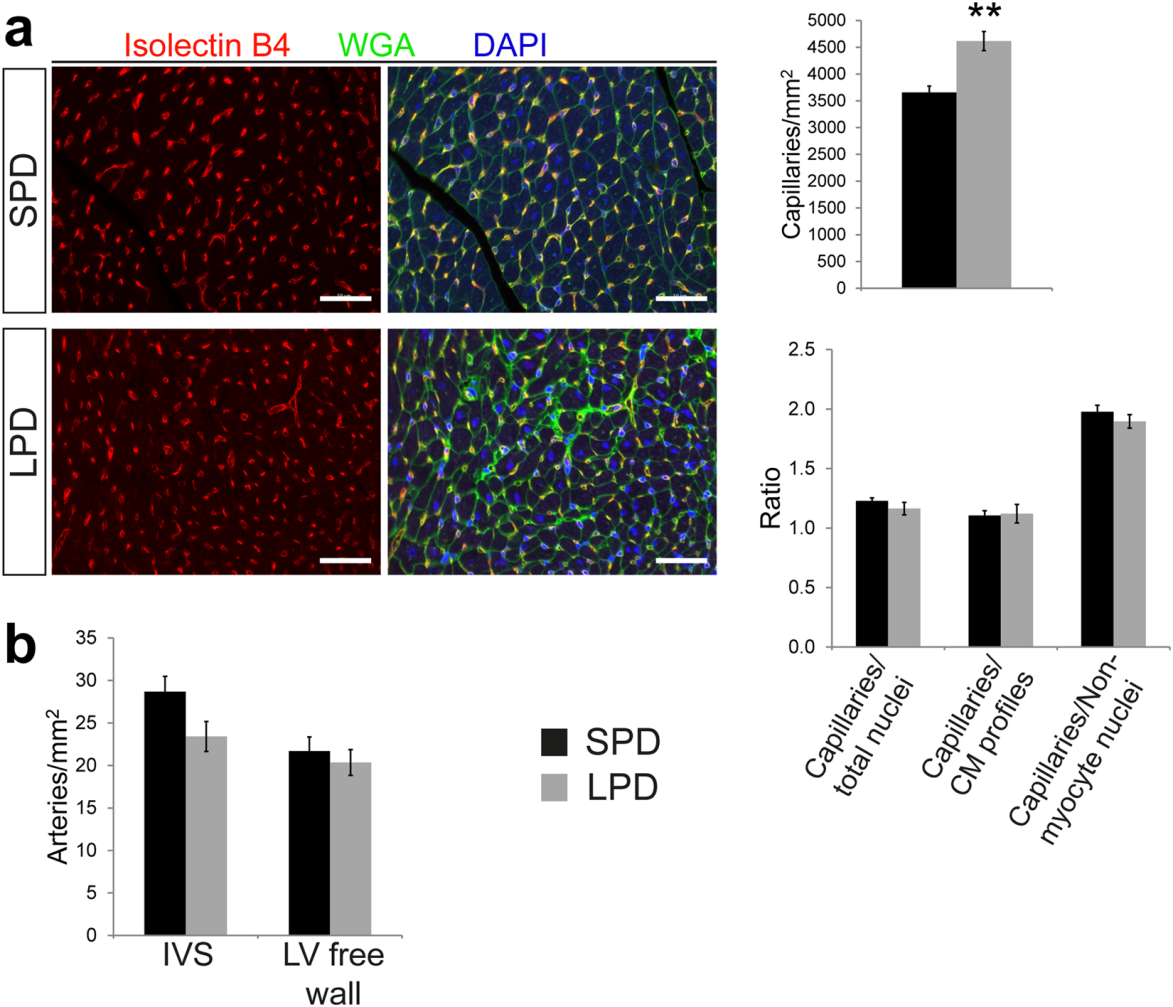
Vascularization of the LV myocardium in adult mice on LPD. (**a**) Detection of capillary profiles in the LV myocardium of adult SPD and LPD hearts by Isolectin B4 staining (co-stained with WGA and DAPI to visualize cell membranes and nuclei, respectively). The number of capillaries per tissue area was increased in LPD versus SPD hearts, whereas the ratio of capillaries per total nuclei, cardiomyocyte (CM) profiles and non-myocyte nuclei was unchanged (SPD n = 6, LPD n = 7 mice, **P < 0.01, scale bar = 50 µm). (**b**) Intramyocardial arteries were detected by immunofluorescence staining for smooth muscle actin and quantified in the LV free wall and interventricular septum (IVS) of adult SPD and LPD hearts. No difference in the number of arteries per tissue area was detected (n = 6 mice per group).

Considering that endothelial cells have recently been proposed to be the most abundant non-myocyte cell population in the adult mouse heart^29^, we hypothesized that LPD might impact on myocardial capillarization. Endothelial cell were detected in tissue sections of adult hearts based on Isolectin B4 staining and quantified in relation to tissue area and cardiomyocyte number (evaluated by WGA/DAPI staining as described in Supplementary Fig. S7 and verified in Fig. 5a). Capillary density per area was increased in LPD compared to SPD adult hearts, but the ratio of capillaries per total nuclear number as well as capillaries per cardiomyocytes was not different between groups (Fig. 6a). Myocardial arteries were detected by SMA staining in vessel walls (Supplementary Fig. S9). The number of SMA positive profiles per tissue area was not different in LPD compared to SPD hearts (Fig. 6b). In conclusion, pre- and postnatal LPD results in increased capillary tissue density but a normal capillary to cardiomyocyte ratio.

### Signaling pathways involved in cell growth and nutrient sensing are not altered in adult hearts after pre- and postnatal LPD

To gain insights into the molecular alterations induced by long-term dietary protein restriction that might underlie the changes in cellular tissue composition in adult female LPD hearts, we performed western blot analyses of key signaling pathways regulating cellular metabolism, nutrient and energy sensing and cell growth. The mTOR (mechanistic target of rapamycin) pathway is regulated by amino acid availability to induce translation and protein biosynthesis required for cell proliferation and cardiomyocyte hypertrophy^30^. mTOR complex 1 activity was normal in adult LPD female hearts, however, evident as unaltered phosphorylation of the well characterized downstream targets S6 ribosomal protein and 4E-BP1 (Supplementary Fig. S10a). Moreover, Ser473 phosphorylation of Akt, a target site for mTOR complex 2^30^, as well as Akt Thr308 phosphorylation was not different between groups (Supplementary Fig. S10a). Similarly, we did not detect altered phosphorylation of p42/44 (ERK1/2) and p38 MAP-kinases in adult LPD hearts (Supplementary Fig. S10b), both of which are involved in cardiac growth^31^ and have been shown to be responsive to amino acid availability^32^.

**Figure 7:**
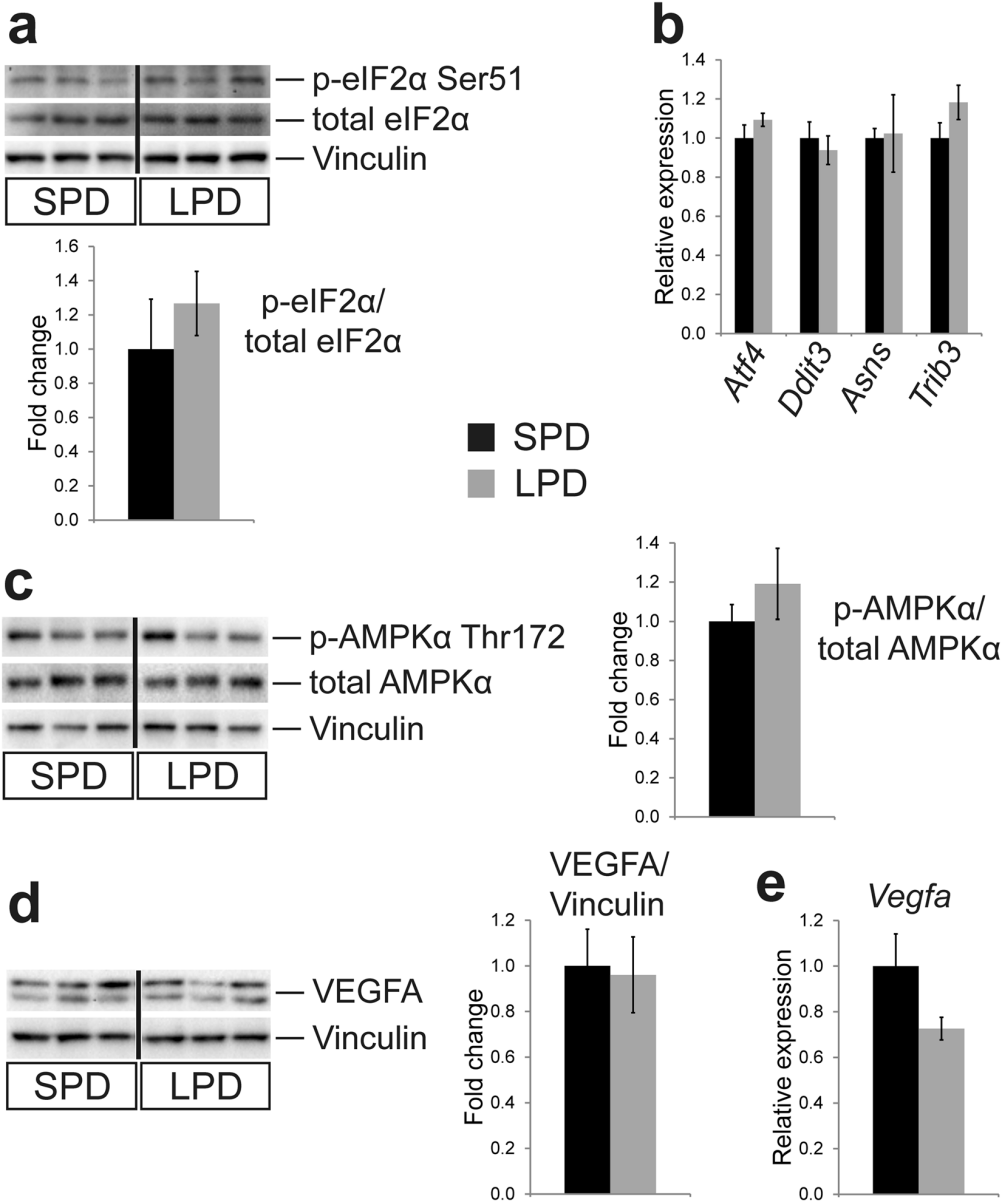
No activation of an amino acid starvation response or induction of VEGF expression in adult LPD hearts. (**a**) Western blot analyses revealed no difference in eIF2α (eukaryotic translation initiation factor 2α) phosphorylation in adult LPD compared to SPD hearts (SPD n = 3, LPD n = 6 litters). (**b**) RNA expression of *Atf4* (activating transcription factor 4) and its target genes involved in amino acid starvation response is not different between SPD and LPD hearts (qRT-PCR data; *Ddit3* = DNA damage inducible transcript 3 (also known as Chop), *Asns* = asparagine synthetase, *Trib3* = Tribbles homolog 3; SPD n = 4, LPD n = 5 litters). (**c**) Phosphorylation of AMPK (AMP activated protein kinase) subunit α is not different between groups (n = 5 litters per group). (**d**) Protein expression of the proangiogenic factor VEGFA (vascular endothelial growth factor A) is not different between SPD and LPD hearts (n = 5 litters per group). (**e**) RNA expression of *Vegfa* is unaltered in adult LPD compared to SPD hearts (qRT-PCR data, SPD n = 4, LPD n = 5 litters). In (**a**), (**c**) and (**d**) samples were run on the same gel but were non-contiguous, as indicated by a vertical black line. Full length, uncropped blots are presented in Supplementary Fig. S11.

It has recently been reported that dietary amino acid restriction increases capillary density in murine skeletal muscle^33^, a phenotype similar to the one seen in heart muscle in this study. The underlying proangiogenic mechanism was proposed to depend on an amino acid starvation response (AASR) involving phosphorylation of eIF2α by GCN2, increased translation of ATF4 and transcriptional induction of VEGF. In addition, activation of AMPK (AMP-regulated protein kinase) results in metabolic adaptations to support endothelial cell proliferation and migration^33^. We did not find evidence that a similar mechanism is active in adult hearts after long-term LPD treatment, as we did not detect differences in eIF2α phosphorylation (Fig. 7a) or RNA expression of ATF4 and AASR target genes (Fig. 7b) between SPD and LPD hearts. Moreover, phosphorylation of the catalytic AMPK subunit α (Fig. 7c) as well as protein and RNA expression of VEGFA was not different between groups (Fig. 7d,e). Our data furthermore excludes an AASR mediated by activation of p42/44 MAP-kinase (ERK1/2) previously reported in cultured cells^34^. In conclusion, long-term dietary protein restriction does not seem to affect some of the key pathways involved in cell growth, energy and nutrient sensing or AASR in the adult heart.

## Discussion

It is well-established that diet impacts on human health and disease. The most striking examples are high caloric diets contributing to the pathogenesis of diseases associated with metabolic syndrome, such as type 2 diabetes, hypertension and coronary heart disease^1–3^. In contrast, caloric restriction positively influences health and lifespan in humans and animal models^9,11^, whereas the specific diet components mediating these effects are under debate. It has been proposed that not caloric intake itself but rather the ratio of dietary macronutrients determines cardiometabolic health, aging and longevity in mice with optimal outcome when dietary protein is replaced by carbohydrates^35^. At the same time low carbohydrate/high protein diets seem to negatively influence cardiovascular health in humans^20,21^. Therefore, dietary protein content has gained increasing attention, as it has been reported that protein restriction reduces overall mortality in young and middle aged humans and mice, whereas high protein intake is essential in the elderly^24^. Further dissecting the role of specific diet components has revealed that sulfur amino acids (methionine and cysteine) mediate some of the beneficial effects of dietary restriction on stress resistance and longevity^36^. The impact of diets on metabolic health is mostly determined at the systemic level (by monitoring body weight, body composition, blood pressure and parameters such as blood glucose, insulin, triglycerides, cholesterol, etc.) whereas changes at the organ or tissue level are less frequently investigated. Our present study suggests that dietary protein restriction positively affects myocardial tissue composition, as mice constantly on LPD had smaller cardiomyocytes, reduced interstitial fibrosis and increased capillary density in the left ventricle compared to SPD controls. Whether the observed changes are influenced by beneficial effects of LPD on body weight, blood pressure or circulating metabolites is unclear at this stage. Also, future studies are warranted to longitudinally follow up hearts constantly on LPD upon ageing and investigate their response to challenges like ischemia or pressure overload. Such analyses will address the possibility that certain myocardial changes upon long-term protein restriction observed in our study might increase stress tolerance of the adult heart thereby promoting cardiovascular health.

Dietary interventions have previously been reported to alter tissue composition in animal models. For example, a high fat diet decreases capillary density in heart and skeletal muscle and increases cardiomyocyte diameter^37,38^. In contrast, caloric restriction accelerates revascularization after hindlimb ischemia in part by increasing capillary density^39^ and exerts proangiogenic functions in cerebral endothelial cells^40^. Importantly, caloric restriction ameliorates myocardial fibrosis in postischemic heart failure and upon ageing as well as age associated cardiomyocyte hypertrophy^14–16^. Similarly, intermittent fasting has positive effects on tissue remodeling after myocardial infarction in rats by attenuating cardiomyocyte hypertrophy, reducing fibrosis and increasing capillary density^17,18^. The impact of dietary protein restriction on myocardial tissue composition is less clear. Maternal LPD during pregnancy is often used in animal studies to induce intrauterine growth restriction and investigate the consequences for disease susceptibility in adulthood^25^. Typically, mothers and pups are switched to standard chow shortly after birth (between postnatal day 1 and 3), such that LPD conditions are effective during pre- and perinatal life. Such diet regime results in maladaptive changes in the adult offspring heart, a pathological gene expression program and increased collagen deposition^41,42^. Dietary protein restriction initiated after birth and maintained throughout the lactation period or LPD started after weaning also negatively affect cardiomyocyte contractility and morphometry as well as myocardial fibrosis^43,44^. Our data show that constant pre- and postnatal LPD reduces cardiomyocyte size and myocardial fibrosis but increases capillary density in the adult heart compared to mice on SPD. Interestingly, LPD restricted to the prenatal period also increases capillary density in the adult heart^41^. A recent study has identified a proangiogenic function of dietary amino acid restriction when initiated in young adult mice resulting in increased capillary density in skeletal muscle^33^. In contrast, prenatal LPD has been proposed to reduce capillary density in adult skeletal muscle^45^. Therefore, although dietary protein restriction could exert certain positive effects on myocardial tissue composition the timing of initiation might be crucial to determine beneficial versus maladaptive changes in the adult heart.

Adult female mice after pre- and postnatal LPD exhibit reduced cardiomyocyte size but normal heart weight compared to mice constantly on SPD. Such counterintuitive findings could be explained by an increase in cardiomyocyte and/or non-myocyte numbers or increased extracellular matrix (ECM) deposition. The latter can be excluded given that LPD hearts exhibit less myocardial fibrosis compared to SPD controls. In contrast, the number of cardiomyocyte nuclei per tissue area was significantly increased in adult LPD myocardium, although this could represent differences in nuclearity rather than cell number^46^. Calculations based on heart weight and cardiomyocyte volume show a trend towards increased cardiomyocyte numbers in LPD compared to SPD hearts, although this difference misses statistical significance. Nevertheless, given that this approach is based on various simplistic assumptions, future studies are warranted to precisely determine cardiomyocyte numbers in the adult LPD heart using more sensitive methodologies, such as stereology or FACS sorting^28,46^. The latter would furthermore allow to more precisely quantify non-myocyte cell populations. Interestingly, cardiomyocyte cross sectional area is increased in LPD female hearts at birth but heart weight is not different compared to SPD controls. This might indicate compensatory hypertrophy to account for a reduced cardiomyocyte number. Prenatal LPD in rats has been reported to cause a cardiomyocyte deficit at birth^47^ which is normalized until weaning^48^. In agreement, others have reported reduced proliferation in the neonatal rat heart upon LPD followed by a boost around day 7^49^. Our data raise the interesting hypothesis that combined pre- and postnatal dietary protein restriction in female mice reduces cardiomyocyte number at birth but through compensatory proliferation in the postnatal period eventually increases cardiomyocyte number in the adult heart, although this will have to be thoroughly tested in future studies.

Our data might have implications for fetal or developmental programming, a phenomenon by which the intrauterine environment impacts postnatal health and disease. Intrauterine growth restriction (IUGR) resulting in low birth weight and small for gestational age babies increases susceptibility towards various chronic diseases in adulthood, including diabetes mellitus, hypertension and coronary artery disease^50^. This is proposed to be caused by alterations of cellular metabolism, gene and protein expression and epigenetics induced in the fetal organism upon unfavorable growth conditions and persisting after birth^50^. According to the thrifty phenotype hypothesis, fetal programming primes the unborn organisms to live in a postnatal environment in which nutrients and resources are likely to be short. In today’s western world, however, a mismatch exists between poor intrauterine growth conditions and a postnatal environment of plenty^51^. It is proposed that after IUGR accelerated postnatal growth during infancy, overfeeding and an unhealthy lifestyle are the main drivers boosting maladaptive consequences of fetal programming^52^. That way, intrauterine adaptations initially intended to be beneficial for the growth restricted baby turn into a risk factor for various diseases in adulthood. Based on this logic, maintaining starvation conditions after birth should prevent fetal programming associated disease after IUGR. In support of such idea, studies in sheep have shown that a mismatch in pre- versus postnatal nutrient conditions induces alterations in blood pressure regulation, renal function and LV morphology that were not observed if nutrient conditions were the same before and after birth^53^. As described above, IUGR can be induced by maternal LPD during pregnancy in mice or rats^25^. Switching pups to standard chow after birth results in maladaptive changes in the adult heart, which also includes contractile dysfunction^25,41,42^. Interestingly, if LPD conditions are maintained for 2 weeks after birth no impairment of heart function was observed in adulthood^54^. Consistently, our data show that constant pre- and postnatal LPD conditions do not adversely affect the offspring´s heart in adulthood under baseline conditions, even though detrimental changes upon cardiac stress or ageing cannot be excluded. This supports the hypothesis that if pre- and postnatal nutrient availability and growth trajectories match, negative effects of IUGR on the adult heart can potentially be prevented or at least delayed. A limitation of our study in regard of such interpretation is that we did not include mice subjected to prenatal LPD but switched to SPD after birth to confirm maladaptive changes in the heart. Interestingly, we applied pre- and postnatal LPD conditions to *cHccs*^+/−^ mice, which are born with smaller hearts due to a reduced cardiomyocyte number and require postnatal compensatory hypertrophy to normalize heart size until adulthood^27^. Heart size and function were normal in adult *cHccs*^+/−^ mice on LPD compared to SPD, indicating that the increased demand for postnatal cardiac growth is not affected by dietary protein restriction and that LPD does not induce cardiac pathology in this mouse model.

After prenatal LPD we observed a reduction in neonatal body weight in female but not male offspring compared to SPD but no changes in heart weight in either sex. This is in contrast to various other IUGR studies that showed reduced body and heart weight in both sexes at birth^25^. This reflects the variety of outcomes reported when using the LPD model, which might depend on species (rats versus mice), diet regime (prior to conception, during pregnancy, postnatally), diet composition, time of analyses (postnatal day 1 versus later stages) and others^25^. Nevertheless, sex specific changes in response to IUGR have been reported previously and fetal programming is known to affect sexes differently^50^. We furthermore observed increased cardiomyocyte cross sectional area in LPD versus SPD females but not males at birth, which could potentially indicate a sex specific effect of LPD on cardiomyocyte growth and proliferation. Interestingly, maternal hypoxia (but not nutrient restriction) during the second half of gestation in guinea pigs reduces the number of cardiomyocytes in the heart of adult female but not male offspring^55^.

A limitation of our study is that the SPD used was a normal rodent chow but not an ideal experimental control diet with the best possible match compared to the LPD. Although this is a general concern in diet induced animal models including protein restriction^56^, it has become clear that factors other than protein content could have confounding effects on the resulting data^57^. The reduction in protein has to be adjusted for by increasing other ingredients (usually fat and starch) to ensure isocaloric conditions. The diets used in this study for example show differences in sugar content (see Supplementary Table S2), such that we cannot exclude that other nutrient components contribute to the observed outcome.

In summary, our data reveal that a combined pre- and postnatal dietary protein restriction does not lead to detectable pathological changes in the murine heart under baseline conditions. In contrast, it is tempting to speculate that myocardial tissue remodeling observed in adult mice permanently on LPD might represent beneficial effects of dietary protein restriction, which could positively influence cardiovascular health and longevity.

## Methods

### Diet composition, treatment regime and heart preparation

Mice were either fed a low protein diet (LPD) containing 8.8% crude protein (ssniff® EF R/M Protein deficient experimental diet, E15202-24) or a standard protein diet (SPD) containing 22% crude protein (ssniff® M-Z complete feed for mice, V1124-3). A detailed diet composition is provided in Supplementary Table S2. Female and male mice were accustomed to LPD conditions for at least two weeks prior to mating and pregnant dams were kept on SPD or LPD during pregnancy. Newborn mice were studied on postnatal day 1 (P1), defined as the morning when litters were first observed. Pups were weight, euthanized by decapitation and hearts were prepared. To study adult hearts, after birth mothers and offspring were kept on the same diet as during pregnancy, offspring were weaned after 3 weeks and remained on their respective diet until early adulthood. At the age of 11 weeks, mice were euthanized by cervical dislocation, weighed and hearts were prepared.

### Mice and sample sizes

Basic considerations for mice used in this study and sample sizes have been described before^26,27,58^. In order to investigate the effect of dietary protein restriction on both normal as well as compensatory postnatal cardiac growth and function, a specific mouse strain which generates a heart conditional *Hccs* (Holocytochrome c synthase) knock-out (KO) was used for all experiments. Mice were kept on a mixed 129 Sv/C57BL6 genetic background. The characterization of pre- and postnatal development in heart conditional *Hccs* KO mice has been described previously^26,27^. Briefly, mice carrying a “floxed” (fl) *Hccs* allele were bred to mice expressing *Cre* recombinase under the control of the *Nkx2.5* promoter. Experiments in adulthood that included heart conditional *Hccs* KO mice were performed on heterozygous *Hccs* KO females (*Hccs*^*fl/wt*^*/Nkx2.5*^*Cre/wt*^, referred to as *cHccs*^+/−^) and their respective female littermate controls (*Hccs*^*fl/wt*^*/Nkx2.5*^*wt/wt*^, referred to as *Hccs*^+/+^). The latter were furthermore used for all other experiments not including *cHccs*^+/−^ females, implying that all mice were carrying the “floxed” *Hccs* allele. Similarly, the genotype of neonates used in the study was *Hccs*^*fl/wt*^*/Nkx2.5*^*wt/wt*^ for all females and *Hccs*^*fl/Y*^*/Nkx2.5*^*wt/wt*^ for all males.

The total number of mice included in the different experiments is as follows: neonates on postnatal day 1: SPD males n = 9 from 5 different litters, SPD females n = 10 from 6 different litters, LPD males n = 7 from 6 different litters, LPD females n = 8 from 6 different litters; adults: SPD *Hccs*^+/+^ females n = 9 from 5 different litters, SPD *cHccs*^+/−^ females n = 9 from 6 different litters, LPD *Hccs*^+/+^ females n = 9 from 5 different litters, LPD *cHccs*^+/−^ females n = 13 from 7 different litters.

For animal studies of developmental programming it is recommended to use only one representative male and/or female per litter in order to compensate for differences in intrauterine or postnatal conditions specific for a particular pregnancy or dam. If more than one mouse per litter is used, this should be accounted for in the statistical analyses^59^. Thus, data was averaged for mice from the same litter if applicable, such that n represents the number of litters included. In case this results in small sample sizes (n ≤ 3), data represent individual mice. Whether n indicates litters or mice is stated in each figure legend.

All animal procedures were performed following institutional guidelines and had previously been approved by the responsible authorities (“Landesamt für Gesundheit und Soziales” (LaGeSo) Berlin, approval number G 0027/10).

### Evaluation of food intake

To measure food intake of adult mice, 5 months old non-pregnant or pregnant females were kept in individual cages. A defined amount of food was weighed and provided to the mice. For non-pregnant mice the remaining food was weighed at the same time each day over the following 10 days and body weight was determined half way through the observation period (i.e. on day 5). For pregnant mice the defined amount of food was provided at 13.5 dpc (days post coitum) and the remaining food was weighed daily until 19.5 dpc. Body weight was determined at the end of pregnancy at 19.5 dpc. Absolute food intake was calculated from the daily decline in food weight and averaged over the observation period (given in gram food per day per mouse) and food intake was furthermore normalized to body weight.

### Echocardiography

Echocardiography was performed as described^58^. Adult mice (11 weeks old) were anesthetized by inhalation of a 2.5% isoflurane/oxygen mixture using the Vevo compact dual anesthesia system (VisualSonics, Toronto, Canada). Body temperature was kept constant at 37 °C using a heat lamp and a rectal temperature probe. Echocardiography was recorded using the Vevo 2100 high frequency ultrasound system (VisualSonics) with a MicroScanTM transducer MS400 set to 30 MHz. Operators were blinded for mouse genotypes and treatment groups during echocardiographic recordings as well as data analyses.

### Histological analyses and immunofluorescence staining

Histology and immunofluorescence staining were performed as described^58^. Hearts were excised, rinsed in cold PBS and fixed in 4% paraformaldehyde/PBS (Sigma Aldrich) for 24–48 hours. The tissue was subsequently dehydrated through an increasing ethanol series, cleared in toluol and embedded in paraffin. 5 µm paraffin sections were stained with hematoxylin & eosin (Carl Roth) to assess overall cardiac morphology or with Sirius Red (Direct Red 80, Sigma Aldrich) to visualize myocardial fibrosis.

For immunofluorescence staining paraffin sections were deparaffinized, rehydrated and heat mediated antigen retrieval was performed in sodium citrate buffer (10 mM, pH 6.0) for 20 minutes. After blocking in antibody solution containing 5% normal goat serum (Jackson ImmunoResearch) for 1 hour, sections were incubated over night with primary antibodies at 4 °C. Secondary antibody detection was performed at room temperature for 1 hour using Alexa Fluor 488 or 555 conjugated secondary antibodies (Life Technologies, 1:500). Nuclei were stained with DAPI or TO-PRO-3 (Life Technologies) and sections were mounted in ProLong Gold antifade reagent (Life Technologies).

### Quantification of myocardial fibrosis

Experiments for quantifying fibrosis have been described previously^58^. Light microscopy images of Sirius Red stained heart sections from 11 week old adult mice were taken using the Biozero BZ-8100 microscope (Keyence, Osaka, Japan). By applying 5x optical magnification, 6 random fields of the left ventricular (LV) myocardium, including free wall and interventricular septum (IVS), were imaged, thereby covering the entire LV tissue. Two non-adjacent cross-sections per heart were used. The percentage of interstitial fibrotic tissue was quantified using the color-threshold plugin of the ImageJ software (https://imagej.nih.gov/ij/), which measures the red stained area in relation to the total LV myocardial area. Values of all 12 images were averaged to give the mean fibrotic tissue content for each heart. Perivascular fibrosis was excluded and deleted from the images prior to analysis.

### Quantification and morphological assessment of coronary arteries

Paraffin sections of postnatal day 1 and adult hearts were stained with an antibody against alpha smooth muscle actin (SMA, Abcam ab5694, 1:200) following standard procedures. For neonates 3 non-adjacent sections per heart and for adults 1 section per heart were included. Neonatal hearts were imaged at 40x and adult hearts at 20x magnification using a Zeiss Axio Scope A1 microscope. SMA positive coronary arteries within the entire myocardium of the LV free wall and interventricular septum were counted irrespective of their size and morphological appearance. The number of coronary arteries was related to the overall tissue area. For size measurements in the neonatal heart, only cross sectioned arteries with a circular profile were outlined along the outer margin of the SMA positive cell layer and the area occupied as well as the outer circumference were measured (using Zeiss ZEN blue software). Approximately 15 arteries per heart were included.

### Evaluation of cellular tissue composition

Paraffin sections of neonatal and adult hearts were stained with Alexa Fluor 488 or 555 conjugated wheat germ agglutinin (WGA, Life Technologies, 1:500) and DAPI to visualize cell membranes and nuclei, respectively. Images of tissue areas with cross sectioned cardiomyocytes within the LV free wall and IVS were captured at 40x magnification using a Zeiss Axio Scope A1 microscope. In neonates 7–10 images and in adults 6–8 images per heart were analyzed. The total number of DAPI stained nuclei per image was automatically counted using the “find maxima” tool of the ImageJ software and manually corrected if necessary. The total tissue area was measured using ZEN blue software (Zeiss). Nuclei were assigned to the cardiomyocyte or non-myocyte cell population based on WGA staining: WGA staining directly adjacent to the nucleus with no visible cytoplasm was considered to indicate non-myocytes, whereas nuclei localized within large cells with a discernable cytoplasmic fringe were scored as cardiomyocytes (see Supplementary Figs S2 and S7). The number of cardiomyocyte and non-myocyte nuclei per tissue area as well as the non-myocyte/cardiomyocyte nuclei ratio was calculated for each image and averaged for each heart. Cardiomyocyte area fraction per image was estimated by measuring the total WGA stained area (which includes cell membranes, non-myocytes, capillaries and extracellular matrix (ECM)) using the “color threshold” tool of the ImageJ software. The WGA positive area (i.e. the non-myocyte/ECM fraction) was expressed as percentage of the overall tissue area and the remaining tissue was considered to represent the cardiomyocyte area fraction.

Cellular tissue composition in the adult heart was validated by staining cardiomyocyte nuclei with an antibody against PCM1 (HPA023374, Sigma Aldrich, 1:200). Secondary detection was performed by Tyramide signal amplification (TSA Plus Cyanine 3 System, Perkin Elmer) according to the manufacturer’s instructions using a horseradish peroxidase (HRP)-linked secondary antibody (Cell Signaling Technology, #7074, 1:500). Nuclei and cell membranes were co-stained as described above.

Fibroblast and myofibroblasts in the adult LV myocardium were detected using immunostaining for vimentin (Abcam, ab7783, 1:100) and smooth muscle actin (Sigma Aldrich, A2547, 1:400), respectively. The number of vimentin and SMA positive cells was manually counted on four confocal laser scanning microscopy (Leica SP5) images per heart and related to the total number of DAPI stained nuclei.

### Evaluation of myocardial capillarization

To visualize endothelial cells in the myocardium, paraffin sections of postnatal day 1 and adult hearts were stained with Alexa Fluor 568 conjugated Isolectin B4 (Life Technologies, I21412, 1:200). Sections were co-stained with Alexa Fluor 488 conjugated wheat germ agglutinin (Life Technologies, W11261, 1:500) and DAPI to visualize cell membranes and nuclei, respectively. Images of tissue areas with cross-sectioned cardiomyocytes within the LV free wall and IVS were captured at 40x magnification using a Zeiss Axio Scope A1 microscope. In neonates 7–10 images and in adults 6–8 images per heart were analyzed using ZEN blue software (Zeiss) and ImageJ. The total number of nuclei as well as the number of cardiomyocyte and non-myocyte nuclei was determined for each image as described above. In adult hearts, the total number of cardiomyocyte profiles (with and without visible nuclei) was furthermore counted. Capillary profiles were manually counted and normalized to the underlying tissue area to indicate capillary density. In neonatal hearts the ratio of capillaries per cardiomyocyte nuclei and in adult hearts the ratio of capillaries per cardiomyocyte profiles and non-myocyte nuclei were calculated. Values were determined for each image and averaged per heart.

### Evaluation of cardiomyocyte size, volume and number

Cardiomyocyte cross sectional area (CSA), length, volume and number per heart were determined as previously described^58^. Briefly, CSA was measured in the LV myocardium based on WGA staining whereas cardiomyocyte length was measured by visualizing intercalated discs based on N-cadherin immunostaining using rabbit polyclonal antibody sc-7939 (1:200) from Santa Cruz (see Supplementary Fig. S5). Mean cardiomyocyte volume per heart was calculated by multiplying mean CSA x mean length. Cardiomyocyte number per heart was calculated based on heart volume, cardiomyocyte area fraction and cardiomyocyte volume^58^.

### Evaluation of cell cycle activity

To assess proliferation rates in neonatal hearts, immunofluorescence staining for phosphorylated histone H3 (p-HH3) was performed (using rabbit polyclonal antibody #9701 from Cell Signaling Technology, 1:200). Sections were co-stained with WGA and DAPI following standard procedures as described above. One longitudinal section per heart was imaged at 40x optical magnification using a Zeiss Axio Scope A1 fluorescence microscope resulting in approximately 10 random fields taken within the LV myocardium (including the free wall and IVS). Cells that exhibited co-localization of p-HH3 and DAPI were considered as cycling. The total number of DAPI stained nuclei as well as p-HH3 positive nuclei were manually quantified using the cell counter plugin of the ImageJ software. For each image the percentage of p-HH3 positive nuclei was calculated. Data from all images were averaged to give the mean proliferation rate for each heart.

### Western blot analyses

Western blot analyses were performed as described previously^58^. For isolation of cardiac protein extracts snap frozen tissue samples derived from apical LV myocardium were homogenized in RIPA buffer supplemented with protease (Complete Protease Inhibitor Cocktail Tablets, Roche Diagnostics) and phosphatase inhibitors (PhosSTOP Phosphatase Inhibitor Cocktail Tablets, Roche) and incubated at 4 °C for 2 hours with gentle agitation. For samples on the same gel equal protein amounts were loaded (40 μg), separated using denaturing polyacrylamide gel electrophoresis (SDS-PAGE) and blotted onto nitrocellulose (GE Healthcare) or PVDF (Millipore) membranes. Membranes were blocked for 1 h in 5% non-fat dry milk (Carl Roth) in TBS-T and incubated with the following primary antibodies at 4 °C over night: phospho-S6 Ser235/236 (#4858, 1:2000), phospho-S6 Ser240/244 (#4838, 1:1000), total S6 (#2217, 1:1000), phospho-4E-BP1 Thr37/46 (#2855, 1:1000), phospho-4E-BP1 Ser65 (#9451, 1:1000), total 4E-BP1 (#9644, 1:1000), phospho-AMPKα Thr172 (#4188, 1:2000), total AMPKα (#2532, 1:1000), phospho-Akt Thr308 (#13038, 1:1000), phospho-Akt Ser473 (#4060, 1:2000), total Akt (#4691, 1:1000), phospho-p42/44 Thr202/Tyr204 (#4370, 1:2000), total p42/44 (#4695, 1:1000), phospho-p38 Thr180/Tyr182 (#4511, 1:1000), total p38 (#8690, 1:1000), phospho-eIF2α Ser51 (#3597, 1:1000), total eIF2α (#2103, 1:1000) (all from Cell Signaling Technology); collagen I (ab34710, 1:5000), vimentin (ab8978, 1:500), osteopontin (ab8448, 1:2000), fibronectin (ab23750, 1:1000), VEGFA (ab46154, 1:1000) (all from Abcam); smooth muscle actin (A2547, 1:2000), α-actinin (A7811, 1:1000) (both from Sigma Aldrich); troponin T (CT3, Developmental Studies Hybridoma Bank, 1:500). Antibodies against GAPDH (MA1-22670, Thermo Scientific, 1:10000), α-Tubulin (T9026, 1:5000) and Vinculin (V9131, 1:5000) (both from Sigma Aldrich) were used for loading control. Secondary detection was performed using horseradish peroxidase (HRP)-linked secondary antibodies (Cell Signaling Technology, #7074 and #7076, 1:2000). Enhanced chemiluminescence (ECL) reaction was performed and detected with the imaging system Odyssey Fc (LI-COR Biosciences) or ChemiDoc XRS + (BIO-RAD). Intensity of detected protein bands was quantified by densitometry using ImageJ software.

### Analyses of RNA expression by qRT-PCR

RNA isolation and qRT-PCR experiments have been described previously^58^. Snap frozen tissue samples derived from apical LV myocardium were homogenized in TRIzol reagent (Invitrogen) and total RNA was isolated according to the manufacturer´s instructions. RNA was subsequently purified using RNeasy spin columns (Qiagen), including digestion of genomic DNA on the column (RNase-free DNase set, Qiagen). Isolated cardiac RNA was reversely transcribed using M-MuLV reverse transcriptase (New England BioLabs) and random hexamer primers. Quantitative real time PCR was performed using the Power SYBR® Green PCR Master Mix (Applied Biosystems) on the ViiA™ 7 real-time PCR system (Applied Biosystems). Primers were obtained from BioTeZ (Berlin, Germany) and sequences are provided in Supplementary Table S3. All primers and PCR conditions were optimized to PCR efficiencies between 90–110% and a correlation coefficient ≥0.990 using cDNA dilution series. All samples were analyzed in triplicates and normalized to GAPDH expression. Relative expression differences between groups were determined using the ΔΔCT method.

### Statistical analyses

Basic considerations for statistical analyses have been described previously^58^. All data were analyzed with SPSS (IBM) or Excel 2010 (Microsoft) software and are presented as mean ± standard error of the mean (SEM). Data sets were tested for normal distribution by Kolmogorov-Smirnov test and homogeneity of variances between groups was assessed by Levene’s test. If these criteria were met, differences between two groups were evaluated with unpaired, two-sided student’s *t*-test and those among multiple groups with one-way analysis of variance (ANOVA) followed by Bonferroni post-hoc test. Differences between multiple groups with unequal variances were evaluated with non-parametric Kruskal-Wallis one-way analysis of variance followed by Mann-Whitney post-hoc test. Neonatal data involving males and females were analyzed by two-way ANOVA with the factors diet, sex and their interaction followed by Bonferroni post-hoc test for pairwise comparison. A probability (*P*) value less than 0.05 was considered to indicate statistical significance (*P < 0.05, **P < 0.01 and ***P < 0.001).

## Supplementary information

Supplementary Information

## Data Availability

All data generated or analyzed during this study are included in this article and its Supplementary Information. No datasets were generated or analyzed during the current study.
