# Supplementary material for: Dietary protein restriction throughout intrauterine and postnatal life results in potentially beneficial myocardial tissue remodeling in the adult mouse heart
Source: Sci Rep. 2019 Oct 22;9:15126. doi: 10.1038/s41598-019-51654-3 (PMC6805892; doi:10.1038/s41598-019-51654-3)
Supplement: Supplementary file 1 — Supplementary Information [file 41598_2019_51654_MOESM1_ESM.pdf]

Supplementary Information

Dietary protein restriction throughout intrauterine and postnatal life results in potentially beneficial myocardial tissue remodeling in the adult mouse heart

Maria Hennig[2], Lea Ewering[1], Simon Pyschny[1], Shinya Shimoyama[1,4,5], Maja Olecka[2], Dominik Ewald[2], Manuela Magarin[2], Anselm Uebing[1], Ludwig Thierfelder[2], Christian Jux[1,3], Jörg-Detlef Drenckhahn[1,2,3]

[1] Department of Pediatric Cardiology, University Hospital Münster, Münster, Germany

[2] Max-Delbrück-Center for Molecular Medicine, Berlin, Germany

[3] Department of Pediatric Cardiology, Justus Liebig University, Gießen, Germany

[4] Department of Pediatric Cardiology, Gunma Children's Medical Center, Gunma, Japan

[5] Department of Pediatrics, Gunma University Graduate School of Medicine, Gunma, Japan

Supplementary Figures

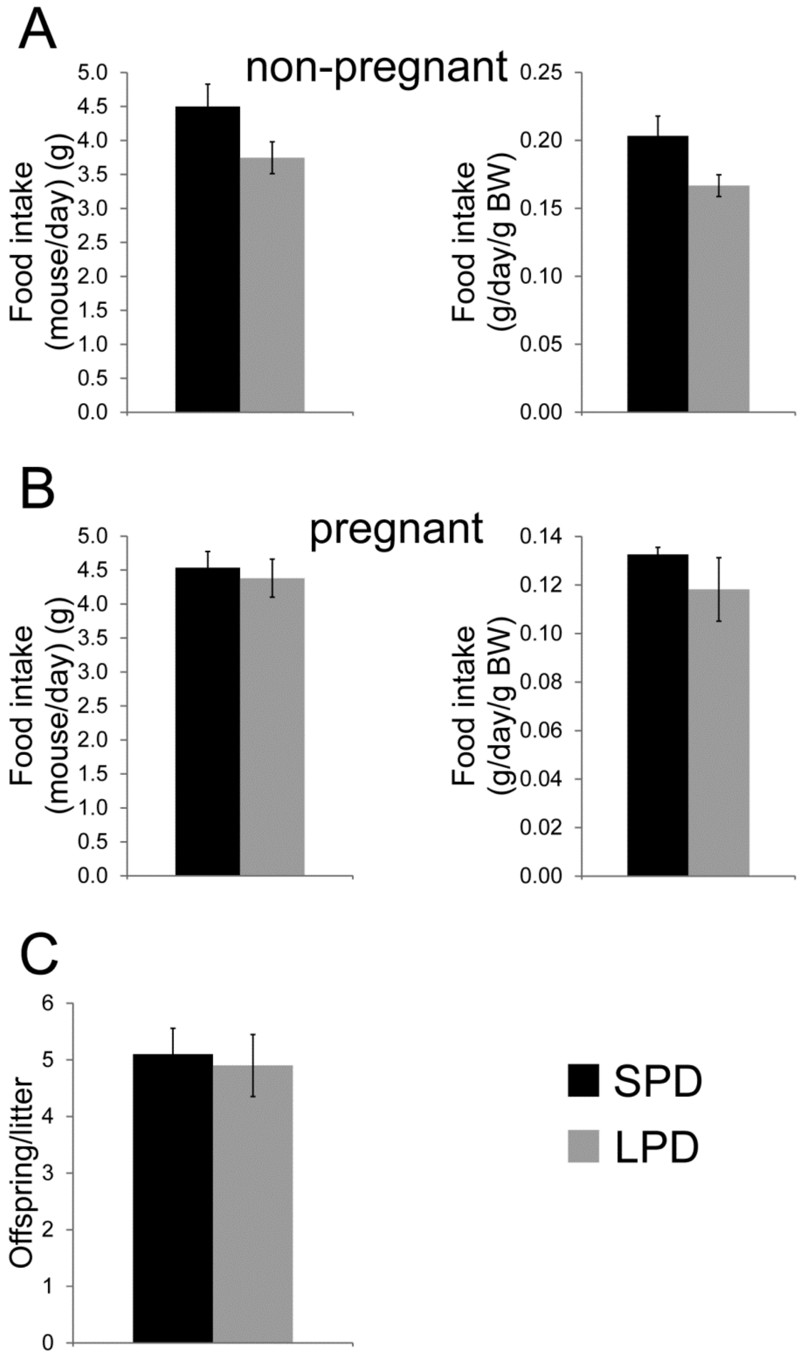

Figure S1. Food intake and litter size in mice on SPD and LPD. (A) Daily food intake was measured in 5 months old non-pregnant female mice on SPD and LPD over a period of 10 days and (B) in 5 months old pregnant female mice from 13.5 dpc until 19.5 dpc. Absolute food intake per mouse per day as well as daily food intake per mouse normalized to body weight is depicted. No significant difference was observed between mice on SPD or LPD (n=3 per group in (A) and (B)). (C) Average litter size of dams on SPD or LPD did not differ on postnatal day 1 (n=10 litters per diet group).

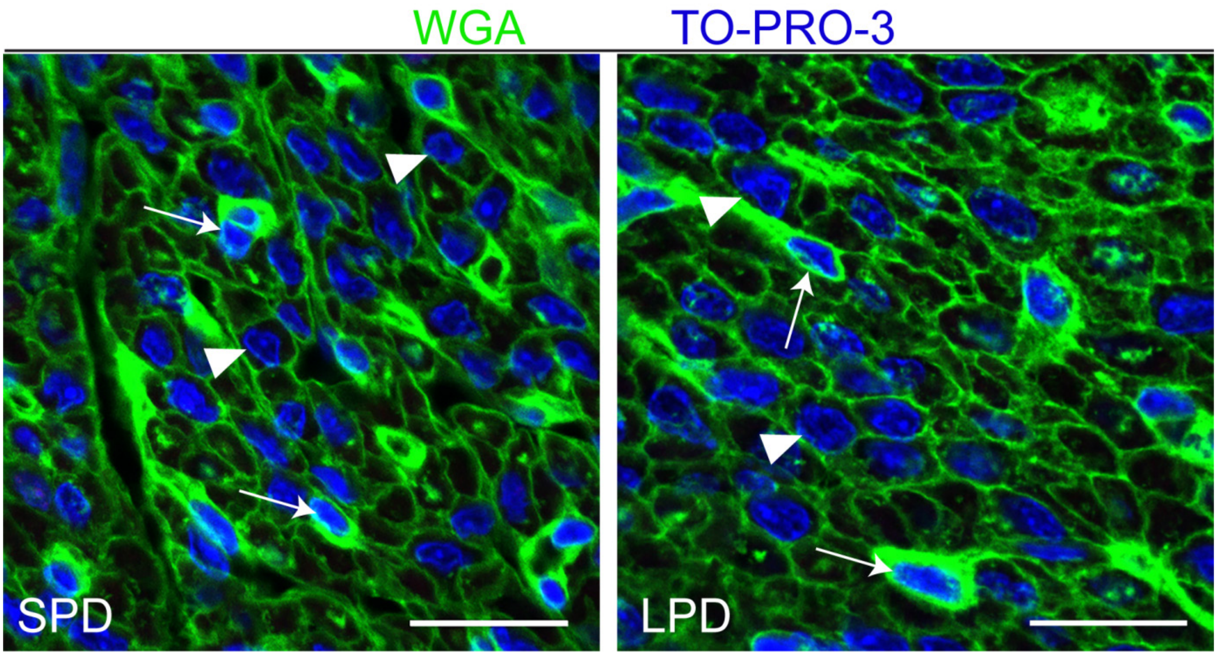

Figure S2. Quantification of cardiomyocyte and non-myocyte nuclei in tissue sections of neonatal hearts. WGA and TO-PRO-3 were used to stain cell membranes (green) and nuclei (blue), respectively. Nuclei were assigned to the cardiomyocyte or non-myocyte cell population based on WGA staining: WGA staining directly adjacent to the nucleus with no visible cytoplasm was considered to indicate non-myocytes (see arrows), whereas nuclei localized within large cells with a discernable cytoplasmic fringe were scored as cardiomyocytes (see arrowheads, scale bar = 20 µm).

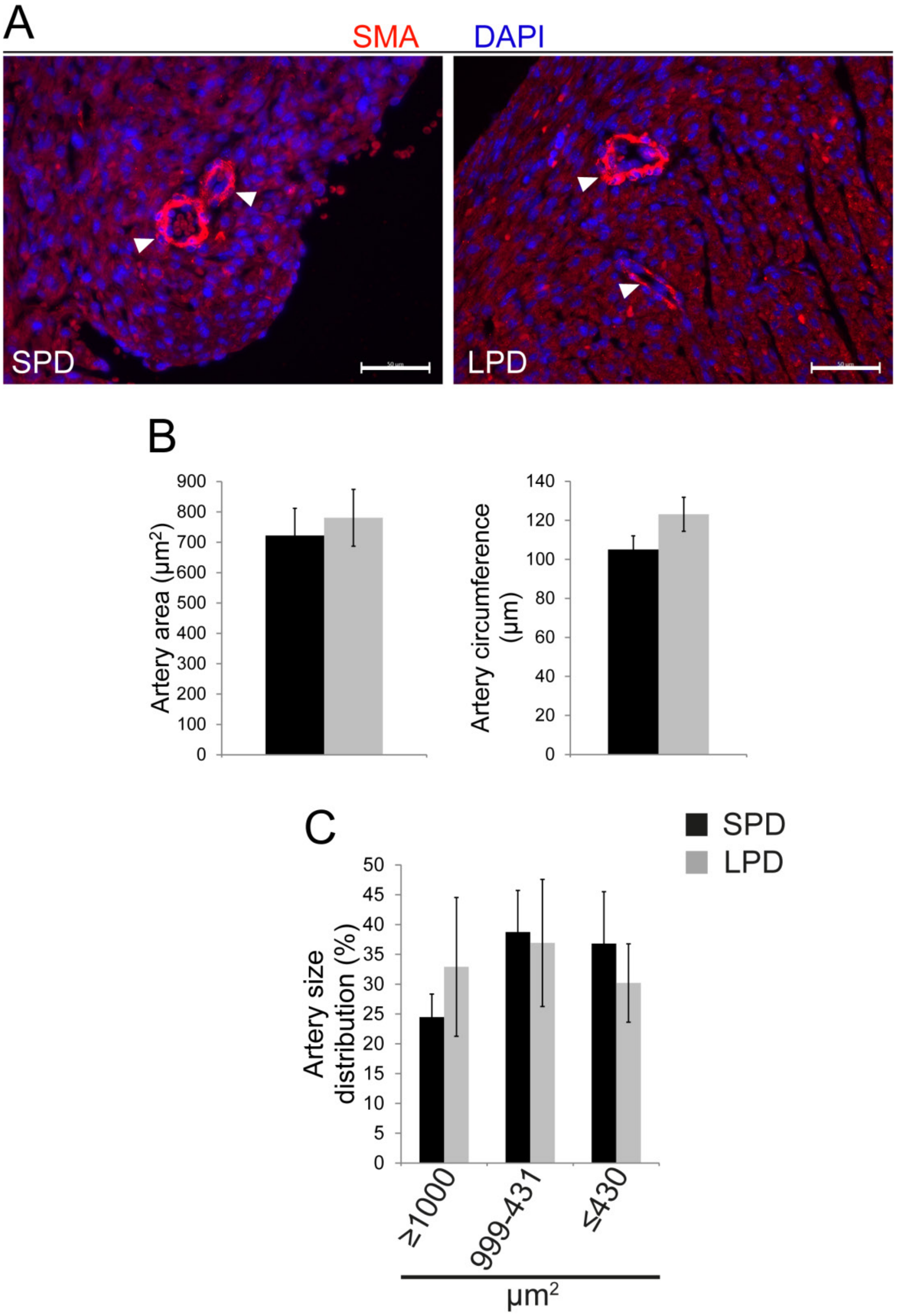

Figure S3. Morphology of coronary arteries within the LV myocardium is not different between SPD and LPD neonates. (A) Coronary arteries within the LV myocardium and interventricular septum were stained with an antibody against smooth muscle actin (SMA, red) in paraffin sections of neonatal hearts. Nuclei were stained in blue using DAPI. Cross sectioned arteries (see arrowheads) were outlined along the outer margin of the SMA positive cell layer and the area occupied as well as the outer circumference were measured (scale bar = 50 µm). (B) The total area and outer circumference of SMA positive arteries did not differ in female hearts from SPD or LPD pregnancies on postnatal day 1. (C) Size distribution of coronary arteries within the LV free wall and IVS of P1 female hearts on SPD or LPD does not differ (n=5 mice per group in (B) and (C)).

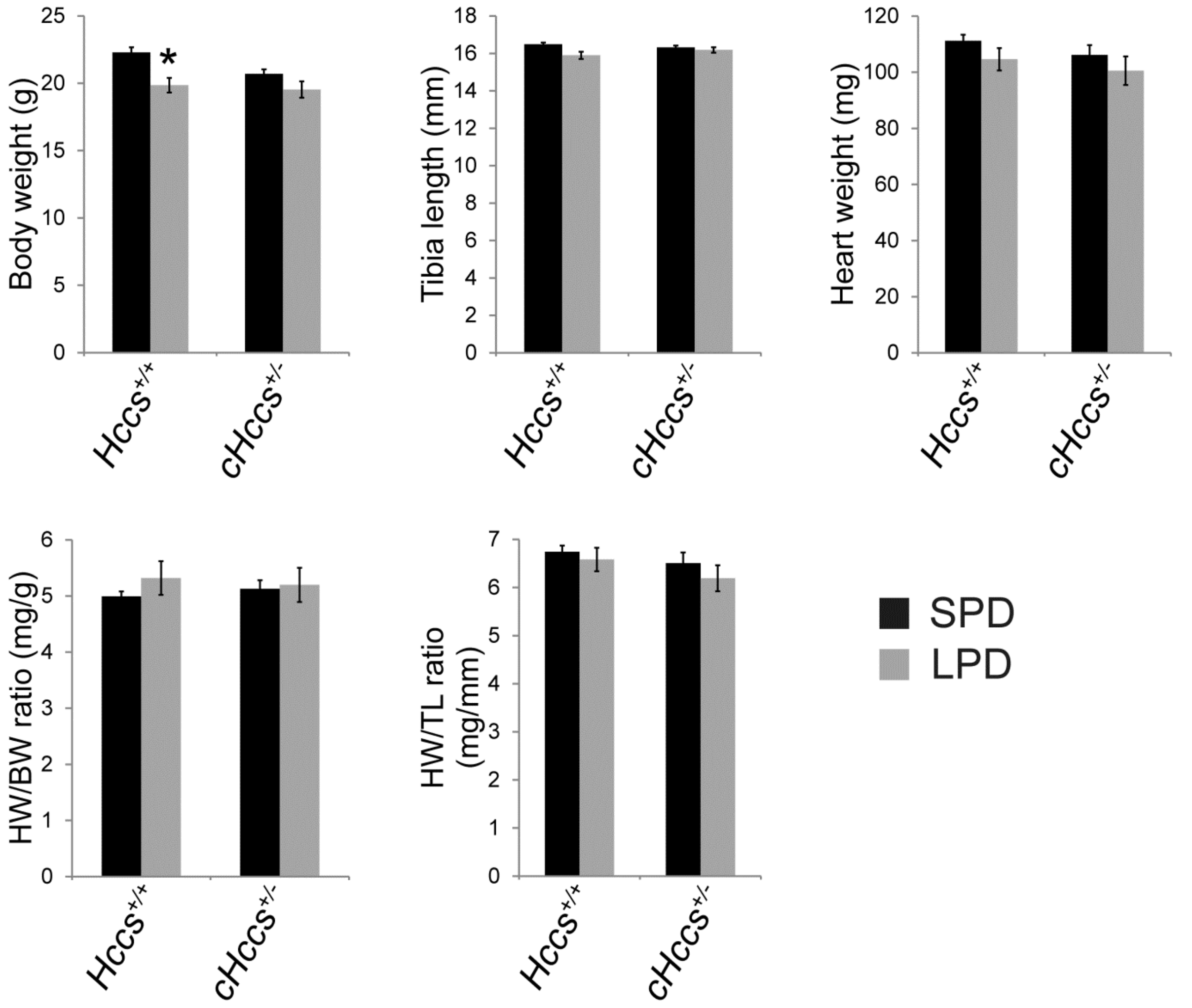

Figure S4. Postnatal normalization of heart weight in *cHccs*$^{+/-}$ female mice is not affected by dietary protein restriction. *cHccs*$^{+/-}$ female mice are born with reduced heart size due to an incomplete cardiomyocyte complement, but heart weight normalizes until adulthood by compensatory cardiomyocyte hypertrophy[27]. Body weight (BW), heart weight (HW) and tibia length (TL) was determined in female mice constantly on SPD or LPD at the age of 11 weeks. Heart weight was furthermore normalized to body weight (HW/BW) as well as tibia length (HW/TL). No differences were observed for any of the parameters between *cHccs*$^{+/-}$ and control mice (*Hccs*$^{+/+}$) or between *cHccs*$^{+/-}$ females on SPD and LPD, indicating that postnatal compensatory growth of the heart in *cHccs*$^{+/-}$ mice is not affected by dietary protein restriction (*Hccs*$^{+/+}$ SPD n=9, *Hccs*$^{+/+}$ LPD n=9, *cHccs*$^{+/-}$ SPD n=9, *cHccs*$^{+/-}$ LPD n=13 mice; **P*<0.05 versus *Hccs*$^{+/+}$ on SPD; note that data presented for *Hccs*$^{+/+}$ females has also been used in Figure 3).

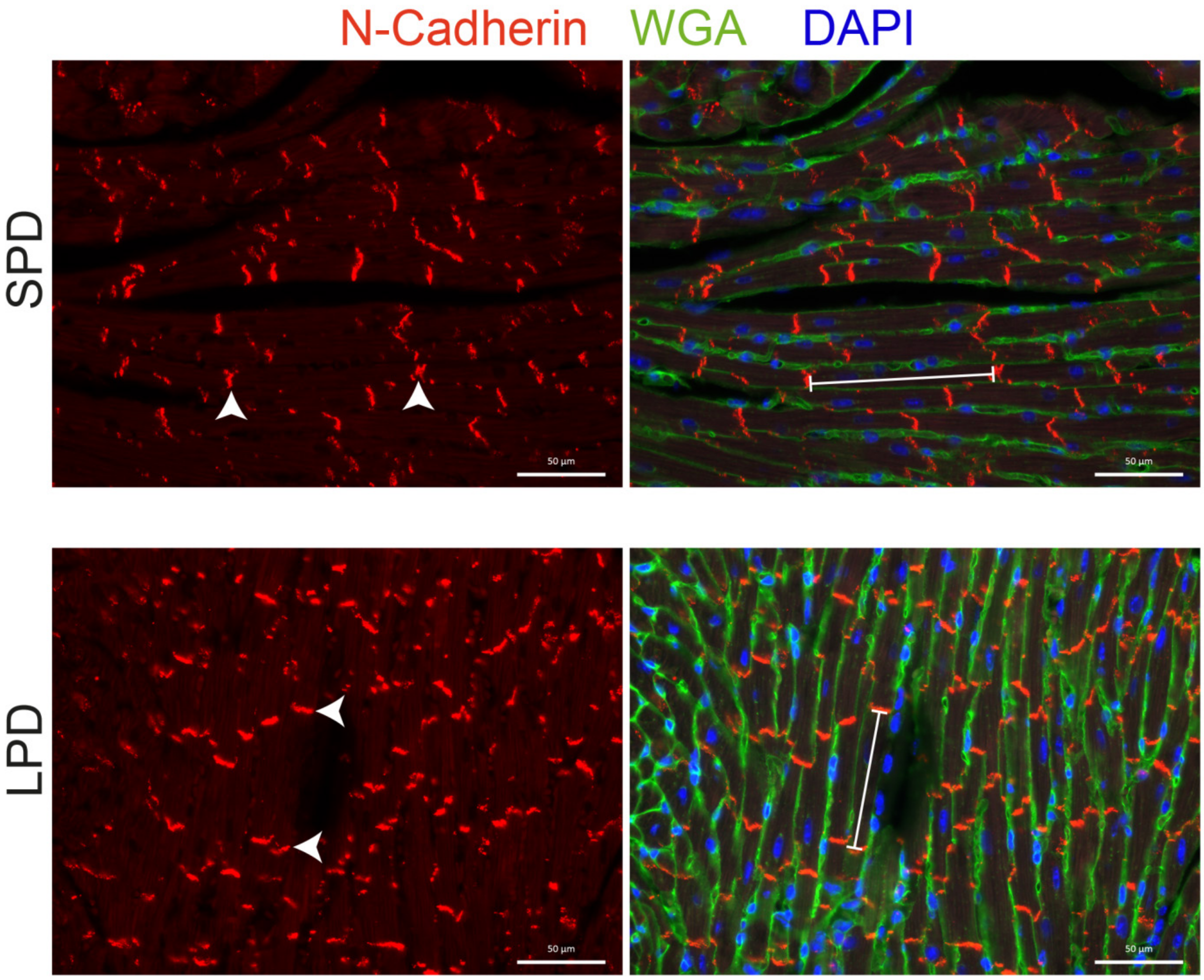

Figure S5. Measurement of cardiomyocyte length in myocardial tissue sections based on N-cadherin staining. Paraffin sections of adult mouse hearts on SPD and LPD were stained with an antibody against N-cadherin to visualize intercalated discs between adjacent cardiomyocytes (red, see arrowheads). Sections were co-stained with wheat germ agglutinin (WGA, green) to mark cell membranes and DAPI (blue) to visualize nuclei. Cell length was measured within the LV myocardium in longitudinally oriented cardiomyocytes with visible nuclei as well as clearly discernible cell membranes and intercalated discs (see examples indicated by white lines, scale bar = 50 µm).

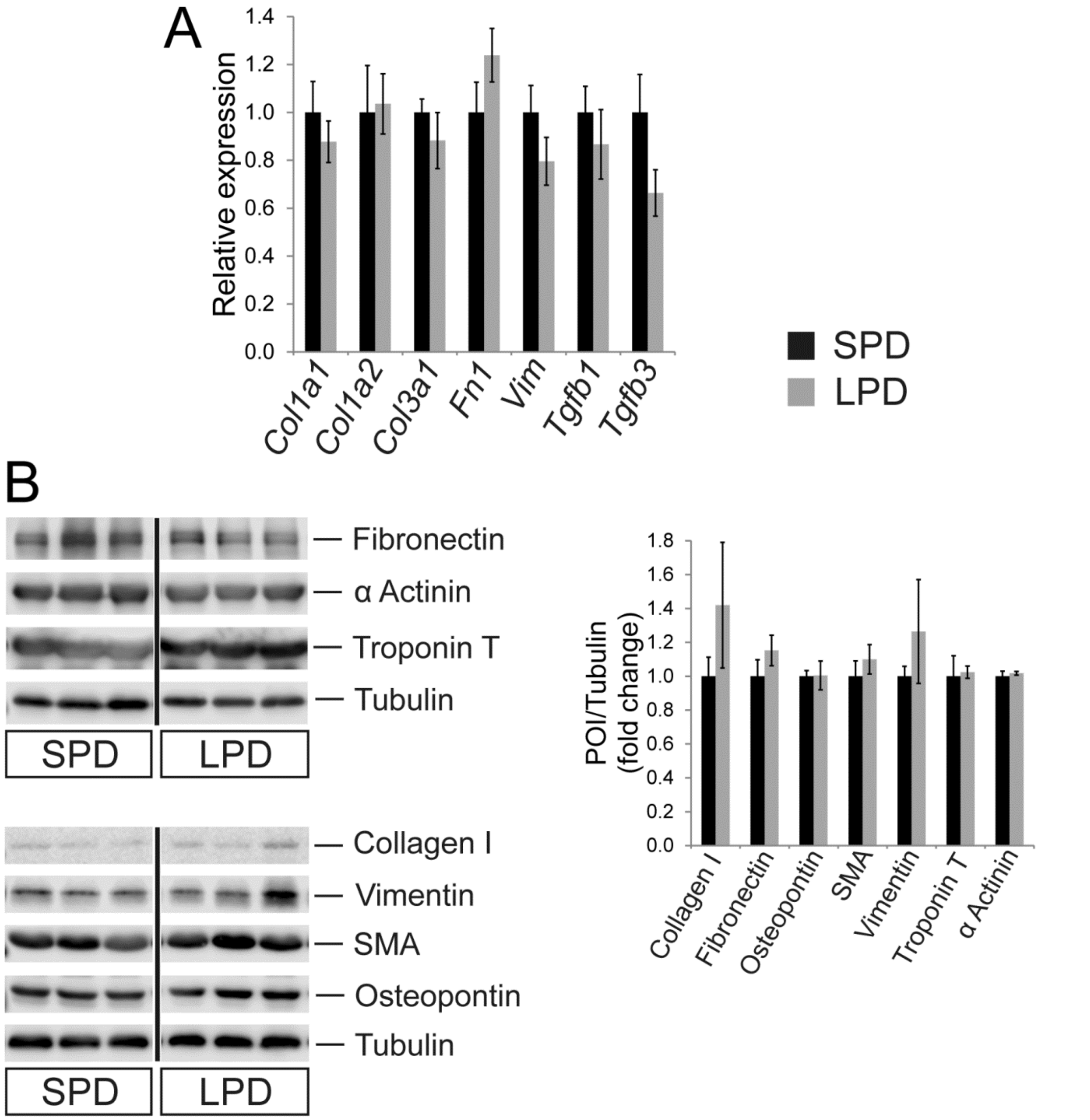

Figure S6. Expression of genes and proteins involved in extracellular matrix composition and myocardial fibrosis in adult SPD and LPD hearts. (A) RNA expression of various genes encoding different collagen components (*Col1a1*, *Col1a2*, *Col3a1*), Fibronectin (*Fn1*), Vimentin (*Vim*), Transforming growth factor β1 and 3 (*Tgfb1*, *Tgfb3*) was evaluated in adult hearts on SPD and LPD by qRT-PCR (SPD n=4, LPD n=5 litters). (B) Western blot analyses of extracellular matrix (Fibronectin, Collagen I) and pro-fibrotic (Osteopontin) proteins, markers for fibroblasts (Vimentin), smooth muscle cells and myofibroblasts (smooth muscle actin, SMA) and proteins involved in cardiomyocyte contractility (α-Actinin, Troponin T) in adult hearts on SPD and LPD. Samples were run on the same gel but were non-contiguous, as indicated by a vertical black line (n=3 mice per group, POI=protein of interest). Full length, uncropped blots are presented in Supplementary Fig. S12.

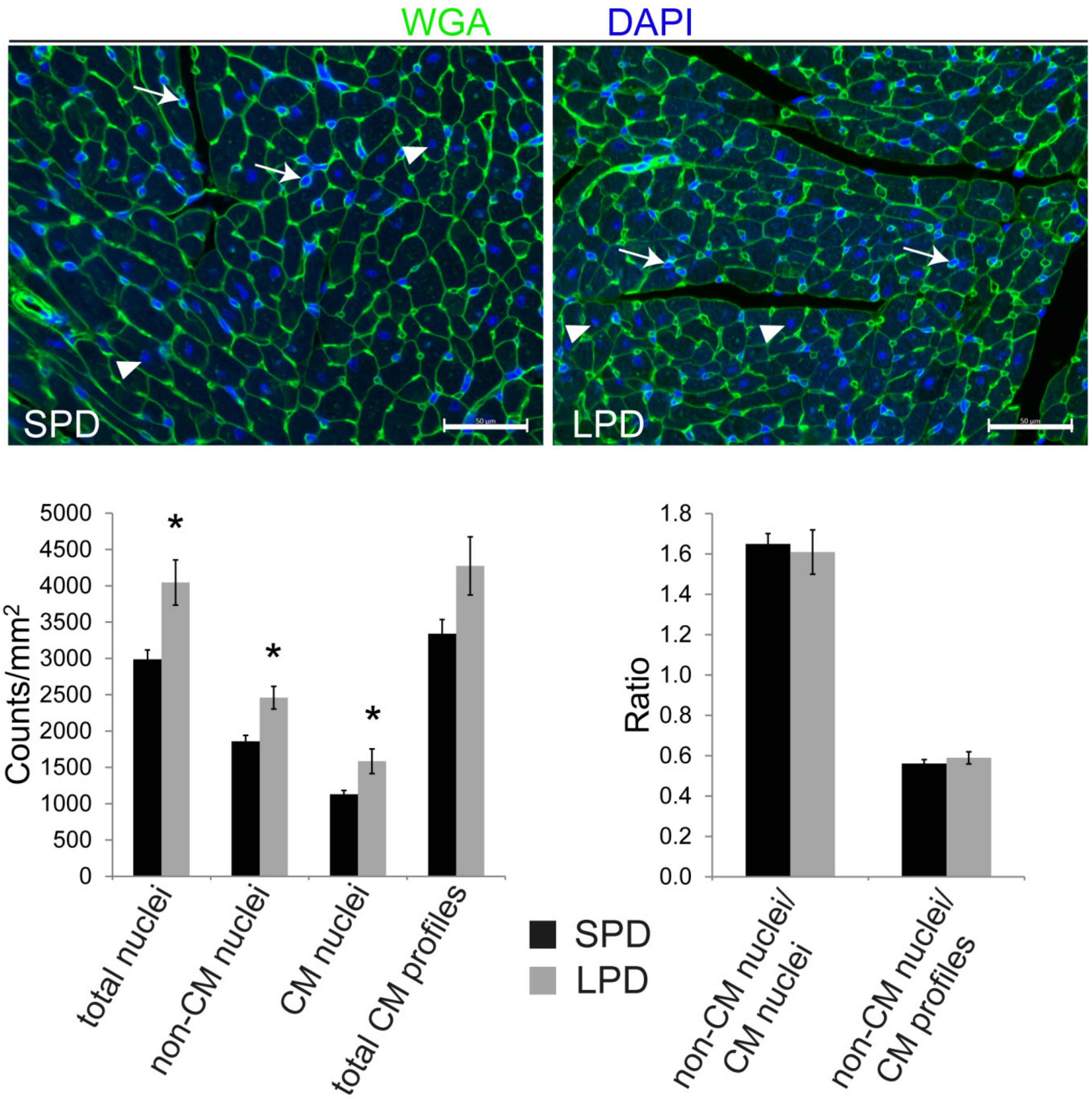

Figure S7. Quantification of cardiomyocyte and non-myocyte nuclei in tissue sections of adult hearts. WGA and DAPI were used to stain cell membranes (green) and nuclei (blue), respectively, in LV tissue sections of adult SPD and LPD hearts. Images were taken in areas of cross-sectioned cardiomyocyte profiles. Nuclei were assigned to the cardiomyocyte or non-myocyte cell population based on WGA staining: WGA staining directly adjacent to the nucleus with no visible cytoplasm was considered to indicate non-myocytes (see arrows), whereas nuclei localized within large cells with a discernable cytoplasmic fringe were scored as cardiomyocytes (see arrowheads, scale bar = 50 µm). The total number of nuclei as well as the number of cardiomyocyte (CM) and non-myocyte nuclei per tissue area was increased in LPD compared to SPD hearts. The total number of cross-sectioned cardiomyocyte profiles (with and without a visible nucleus) was slightly increased in LPD hearts but missed statistical significance (P=0.092). The ratio of non-myocyte/cardiomyocyte nuclei and non-myocyte nuclei/cardiomyocyte profiles was not different between groups (SPD n=3, LPD n=5 litters, *P<0.05).

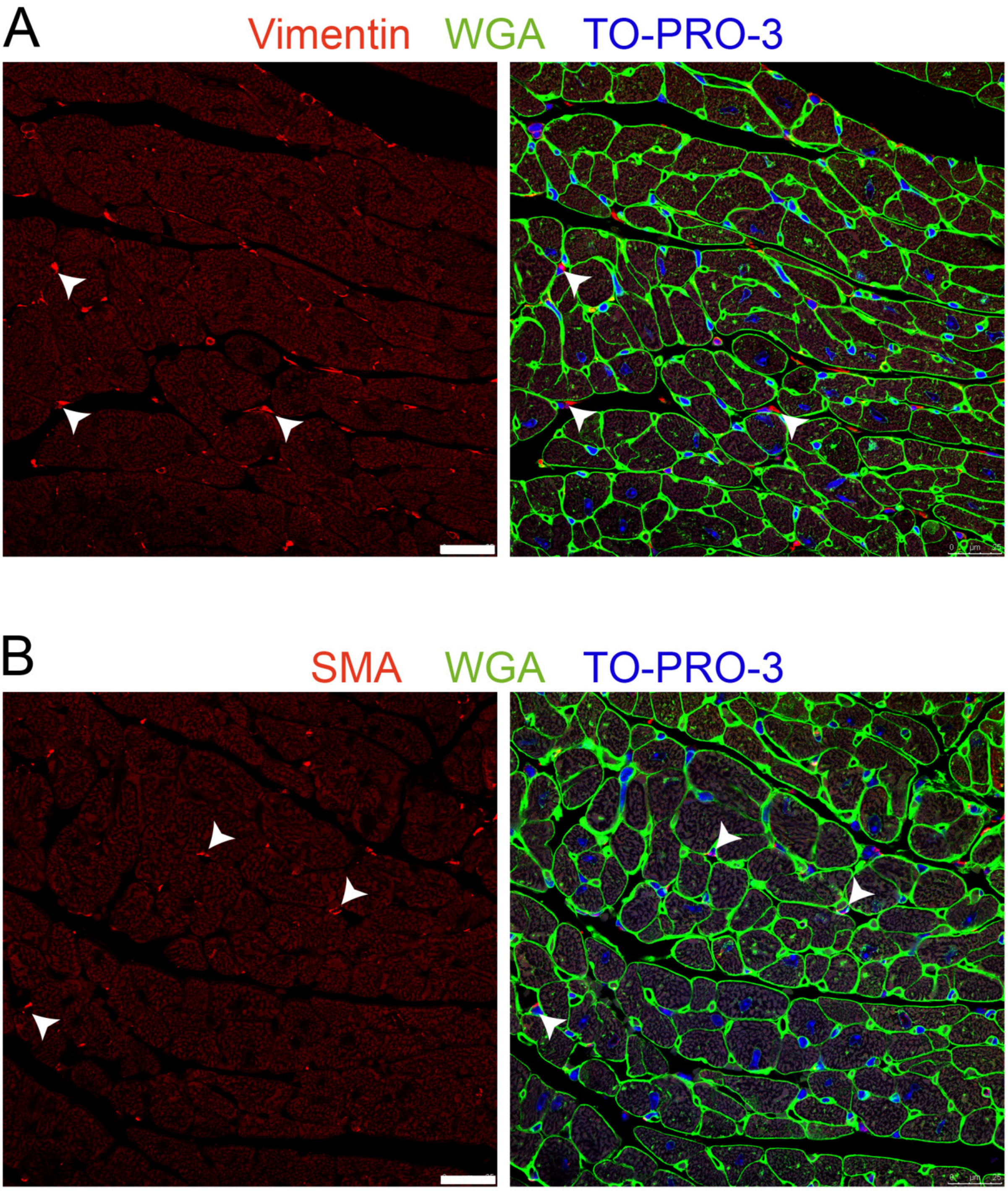

Figure S8. Detection of fibroblast and myofibroblasts in the LV myocardium of adult mice on SPD and LPD. (A) Representative images of the immunofluorescence detection of Vimentin positive fibroblasts and (B) smooth muscle actin (SMA) positive myofibroblasts in the LV myocardium of an adult SPD mouse heart. Vimentin and SMA are shown in red, whereas cell membranes are stained in green using wheat germ agglutinin (WGA) and nuclei are stained in blue using TO-PRO-3. Fibroblasts and myofibroblasts, respectively, were identified as small interstitial cells located between cardiomyocytes (see arrowheads, confocal laser scanning microscopy, scale bar = 25 µm).

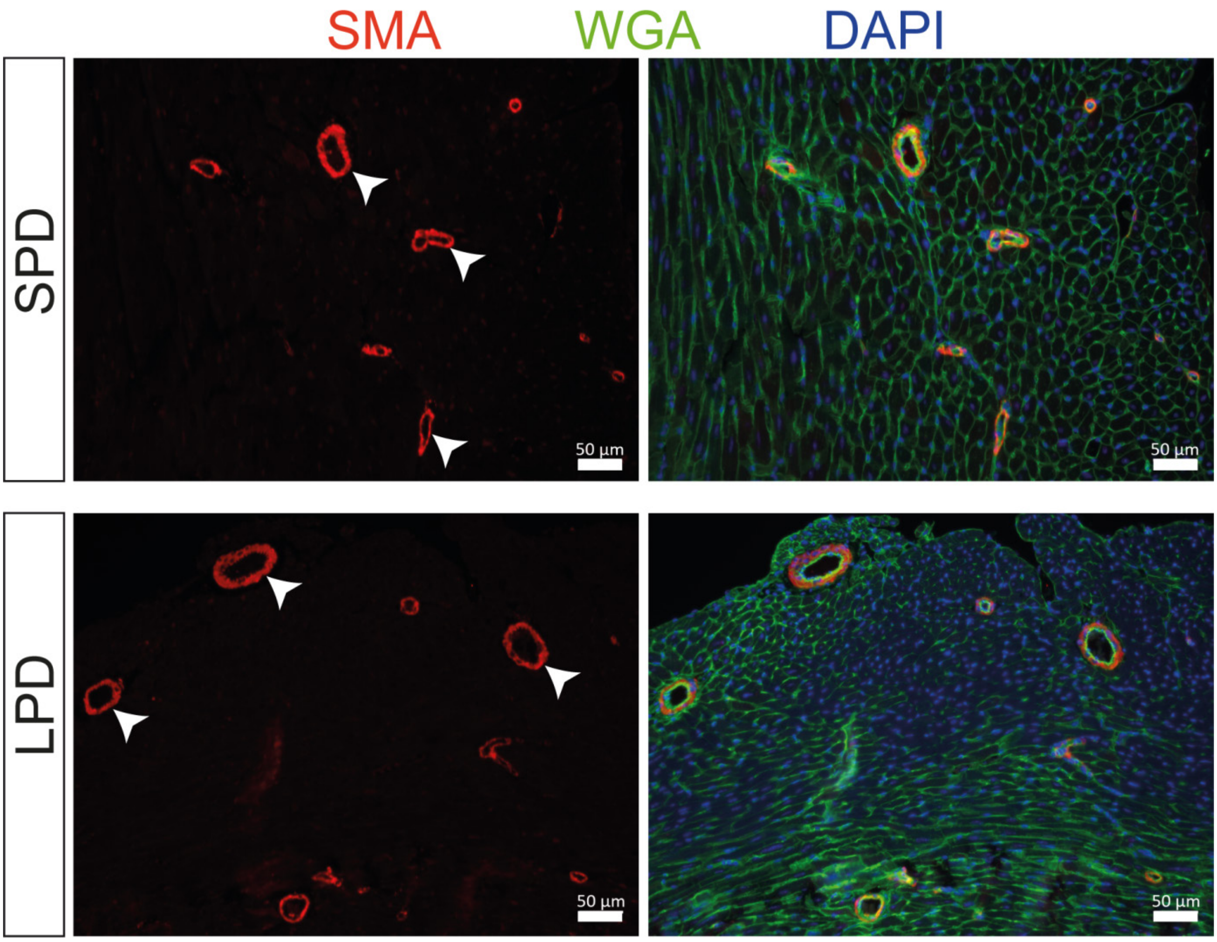

Figure S9. Detection of coronary arteries within the LV myocardium of adult SPD and LPD mice. Representative images of immunofluorescence staining for smooth muscle actin (SMA, shown in red) in the vessel walls of coronary arteries located within the LV myocardium of SPD and LPD adult hearts. Sections were co-stained with WGA (depicted in green) to visualize cell membranes and the endothelial lining of coronary arteries, whereas nuclei were stained in blue using DAPI. Arrowheads indicate examples of circular SMA positive structures with endothelial lining that were scored as intramyocardial arteries (scale bar = 50 µm).

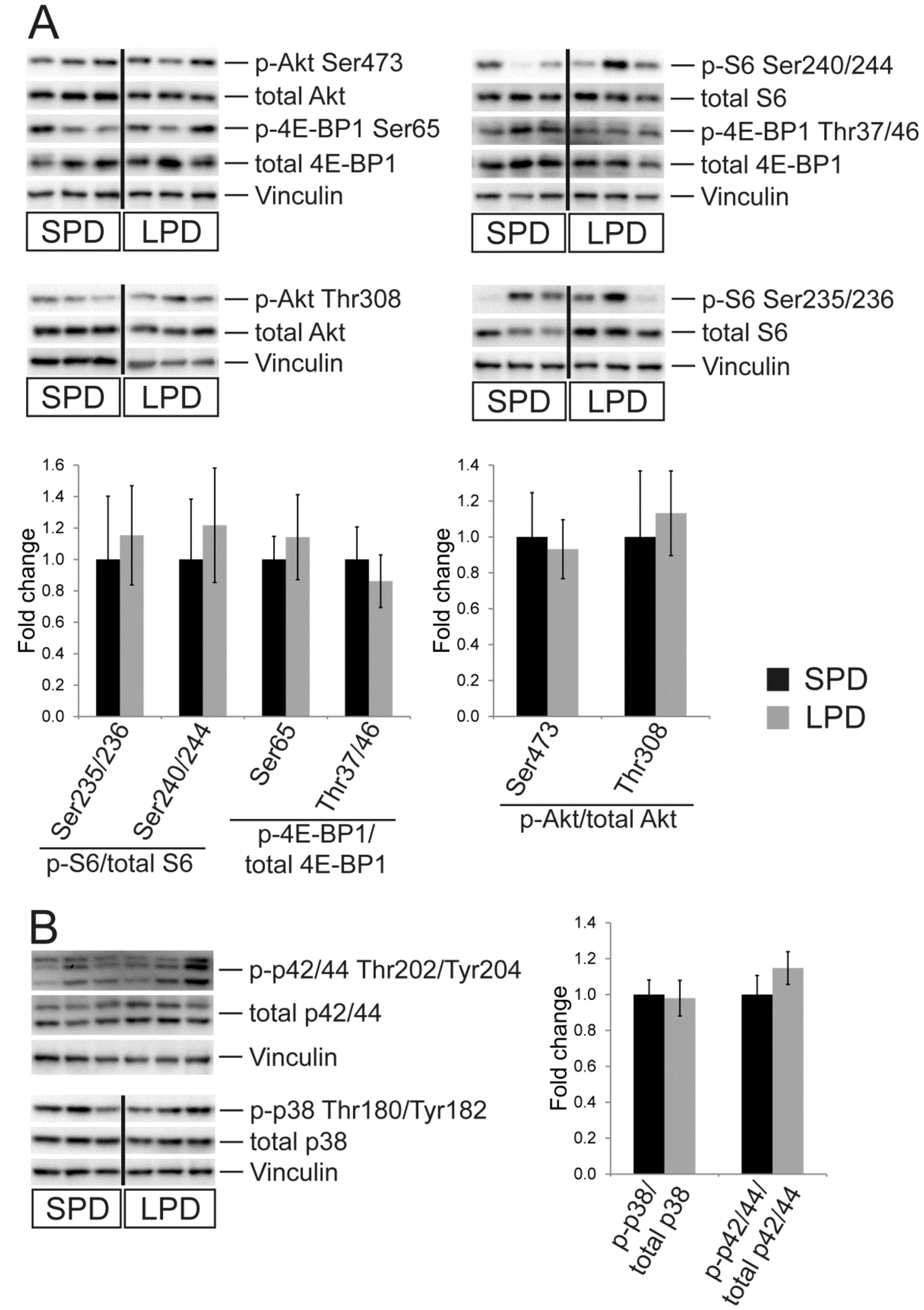

Figure S10. No alterations in key signaling pathways regulating cell growth in adult LPD compared to SPD hearts. (A) Western blot analyses of the mTORC1 (mammalian target or rapamycin complex 1) downstream targets S6 ribosomal protein and 4E-BP1 (eukaryotic translation initiation factor 4E binding protein 1) revealed normal phosphorylation in adult LPD compared to SPD hearts. Similarly, phosphorylation of Akt at Ser473 (mTORC2 target site) and Thr308 (PDK1 target site) was not different between groups. (B) Phosphorylation of the MAP kinases p42/44 (Erk1/2) and p38 was not different between SPD and LPD hearts (n=5 litters per group in (A) and (B)). If samples were run on the same gel but were non-contiguous, this is indicated by a vertical black line. Full length, uncropped blots are presented in Supplementary Fig. S13 – S15.

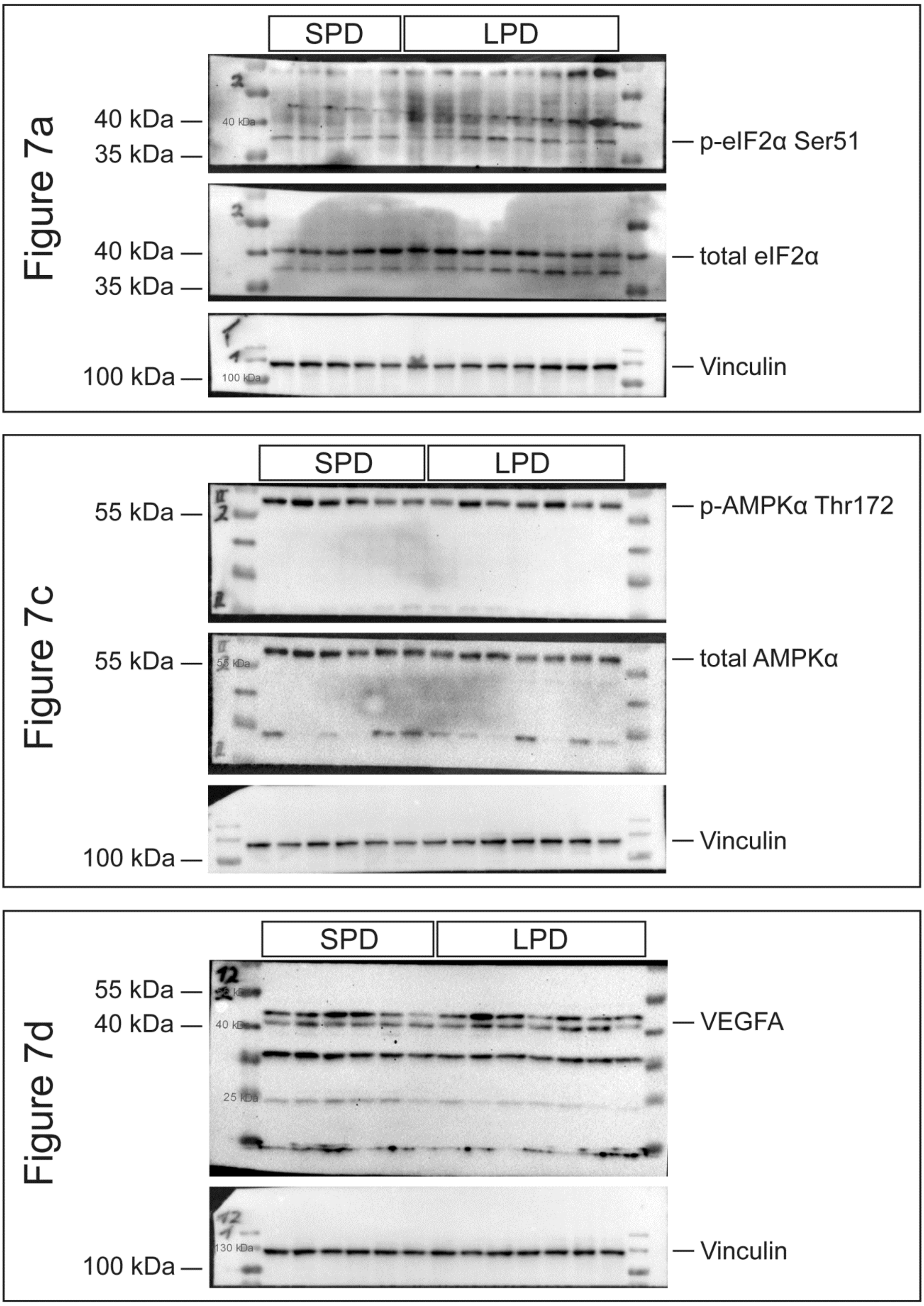

Figure S11. Uncropped western blots corresponding to Fig. 7.

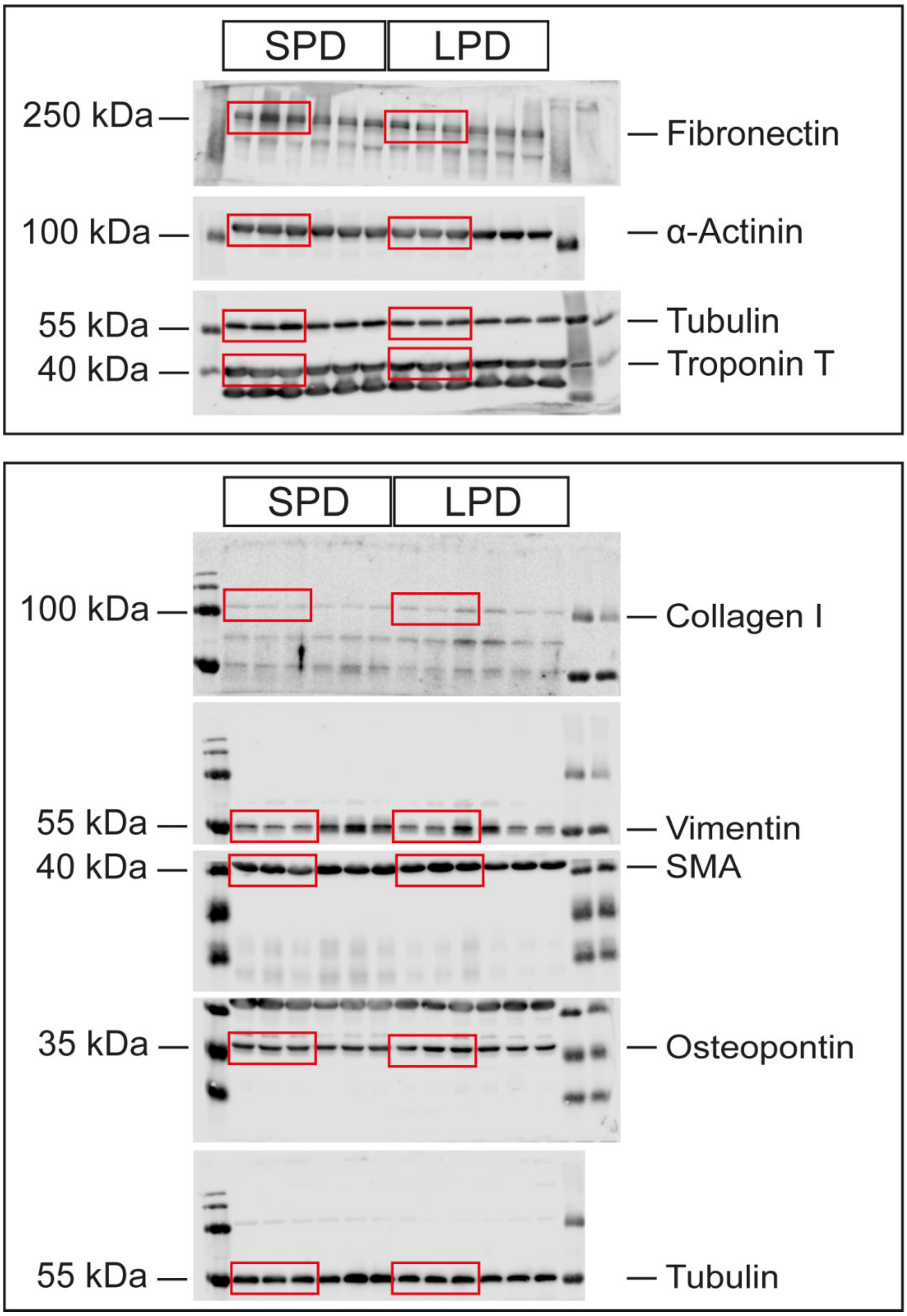

Figure S12. Uncropped western blots corresponding to Supplementary Fig. 6.

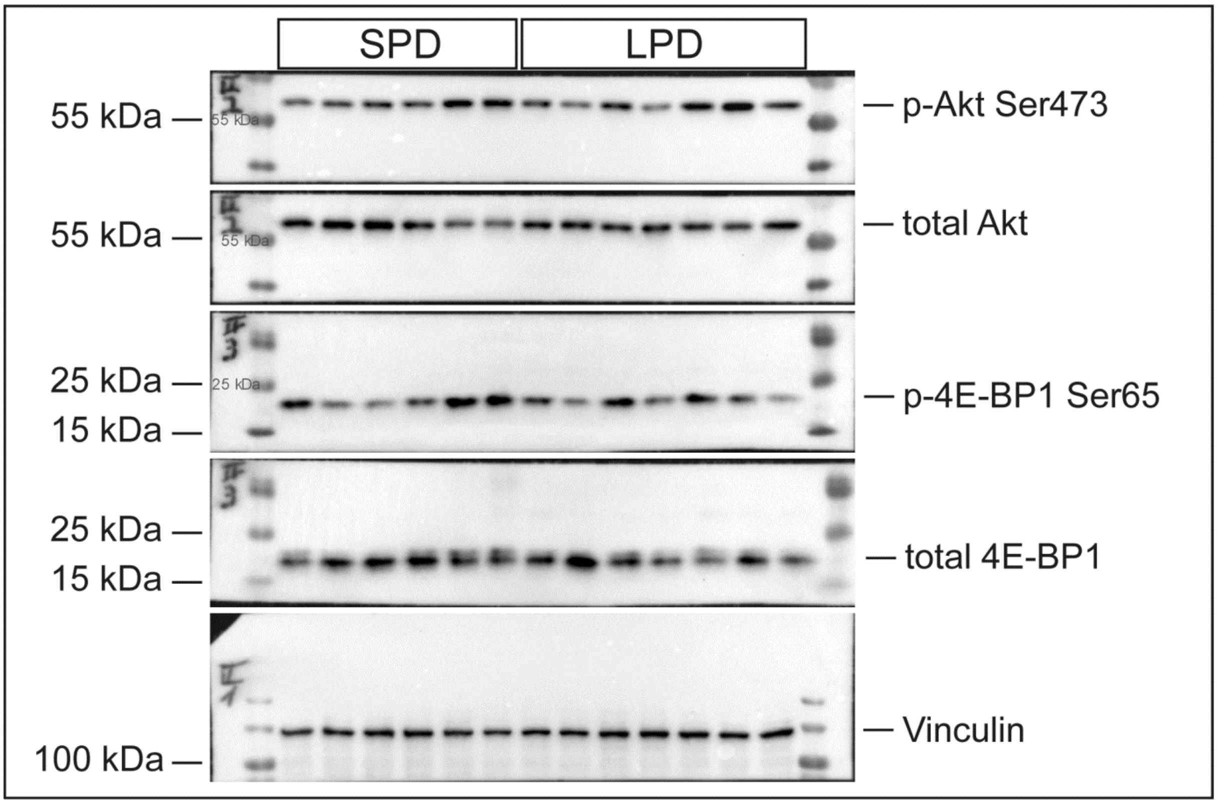

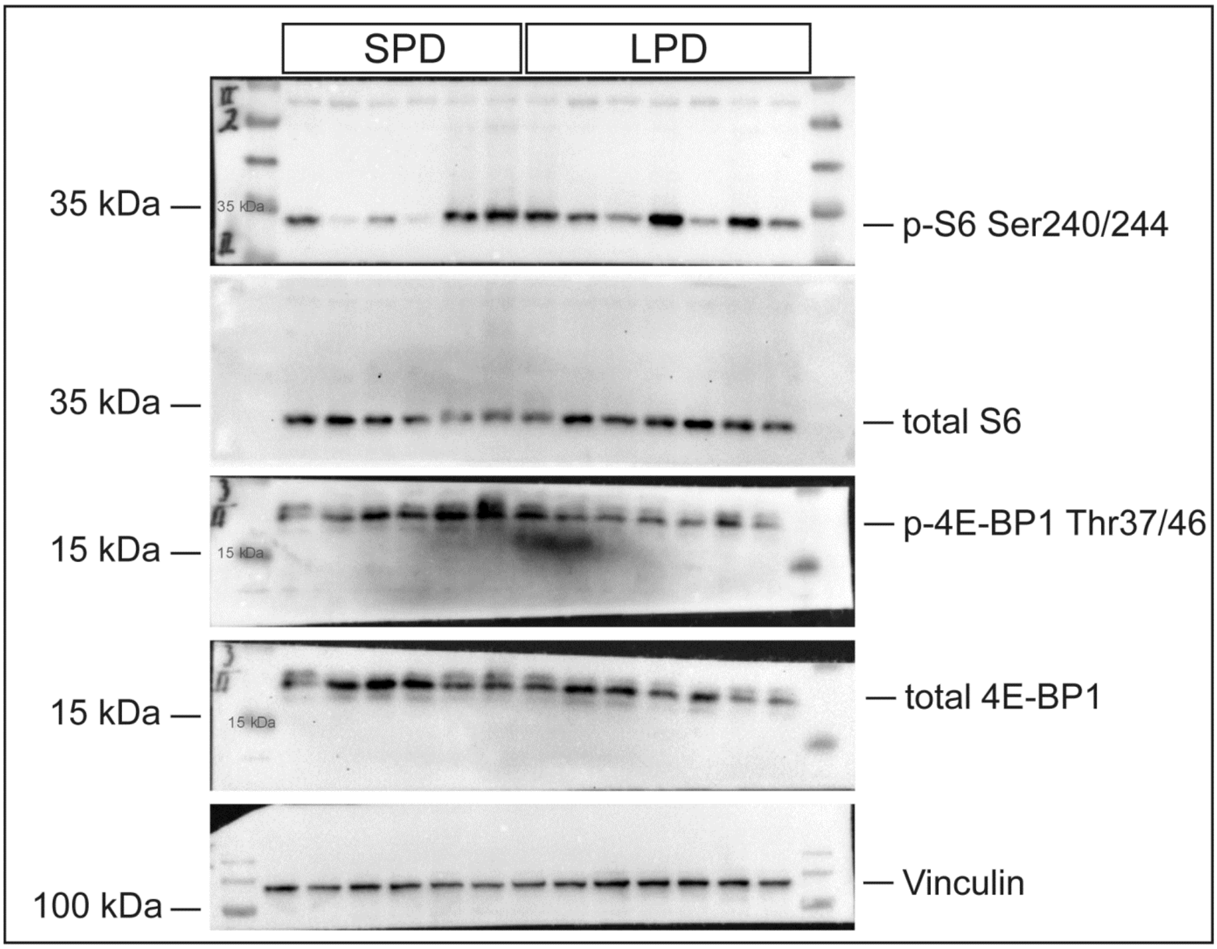

Figure S13. Uncropped western blots corresponding to Supplementary Fig. 10A.

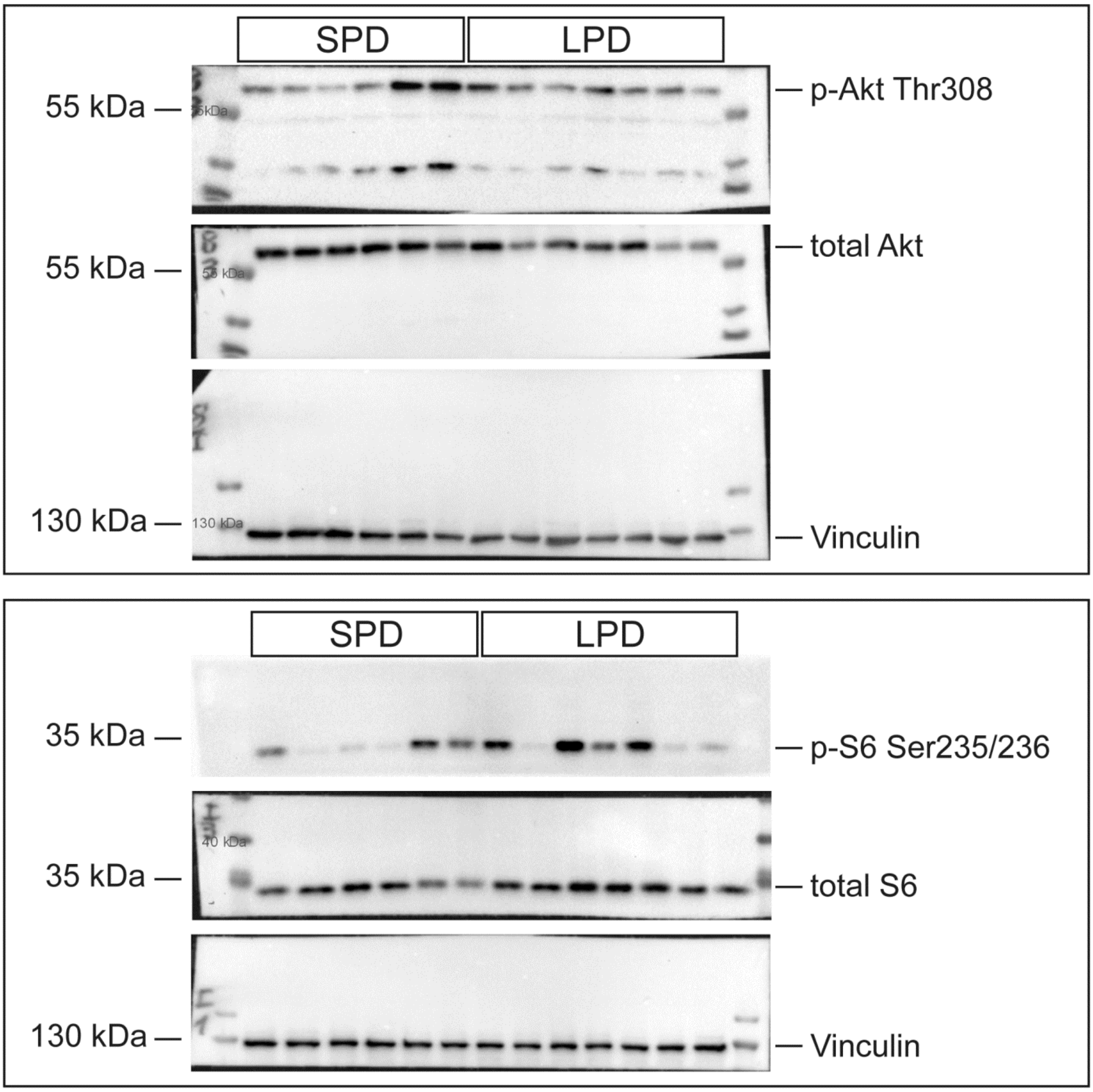

Figure S14. Uncropped western blots corresponding to Supplementary Fig. 10A.

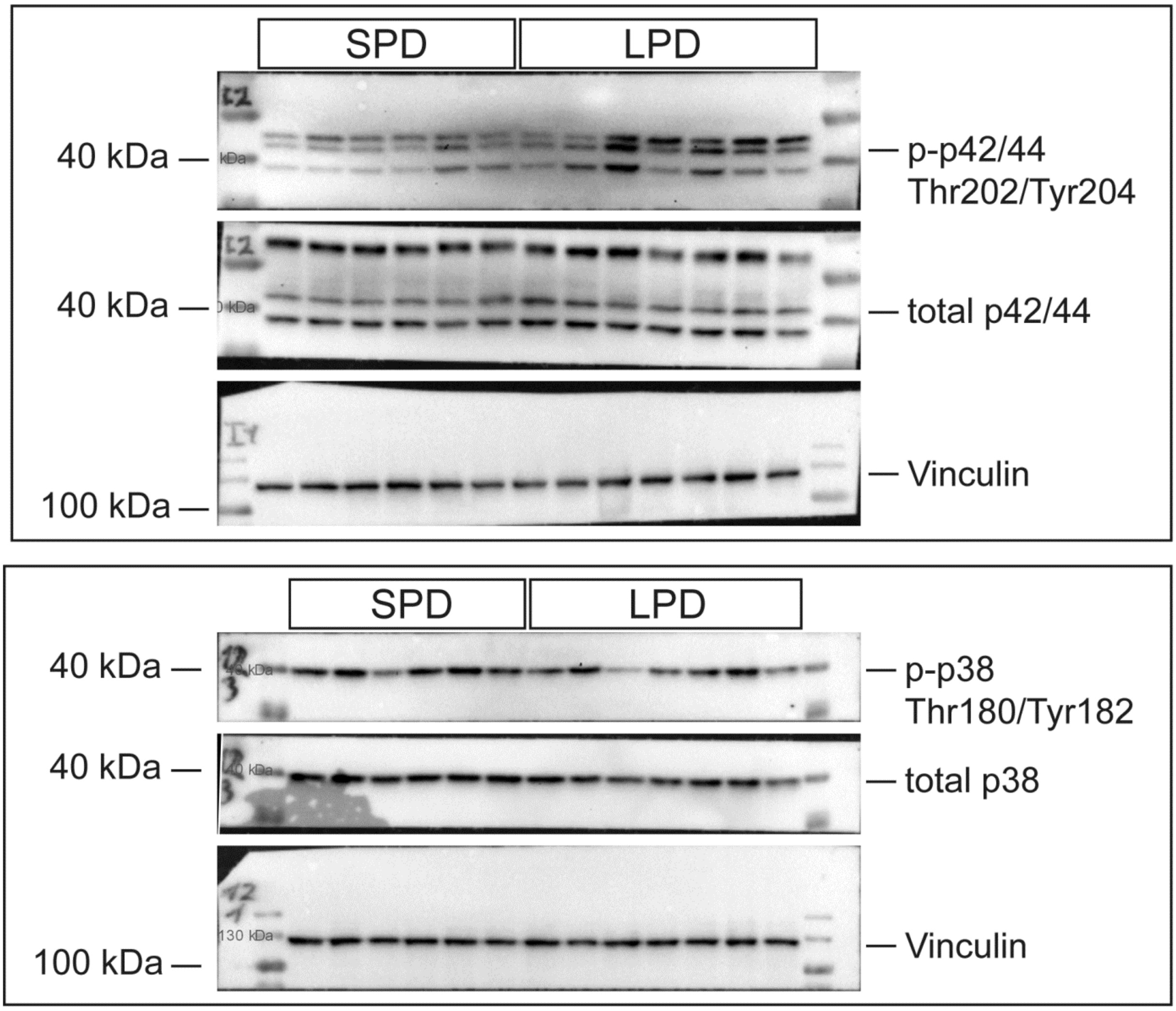

Figure S15. Uncropped western blots corresponding to Supplementary Fig. 10B.

Supplementary Tables

Table S1: Echocardiographic measurements in adult control (*Hccs*$^{+/+}$) and heart conditional *Hccs* knockout (*cHccs*$^{+/-}$) mice on SPD and LPD.

	n=	IVS dia (mm)	LVPW dia (mm)	IVS sys (mm)	LVPW sys (mm)	LVID dia (mm)	LVID sys (mm)	FS (%)	EF (%)
**SPD**									
Hccs$^{+/+}$	7	0.66 ±0.03	0.65 ±0.03	1.05 ±0.07	1.00 ±0.07	3.96 ±0.07	2.74 ±0.17	31.03 ±3.23	59.12 ±4.50
cHccs$^{+/-}$	6	0.71 ±0.02	0.69 ±0.03	1.06 ±0.04	0.99 ±0.04	4.01 ±0.09	2.91 ±0.13	27.71 ±1.90	53.09 ±3.51
**LPD**									
Hccs$^{+/+}$	6	0.72 ±0.03	0.74 ±0.02	1.05 ±0.05	1.05 ±0.05	3.70 ±0.06	2.62 ±0.20	29.38 ±4.56	56.23 ±7.33
cHccs$^{+/-}$	7	0.77 ±0.04	0.75 ±0.05	1.05 ±0.04	1.03 ±0.04	3.68 ±0.13	2.69 ±0.23	27.71 ±4.21	53.64 ±5.31

Echocardiography was performed in 11 week old *cHccs*$^{+/-}$ and control *Hccs*$^{+/+}$ female mice on SPD or LPD. Left ventricular (LV) wall thickness and diameter were determined in systole (sys) and diastole (dia) and contractility was calculated. IVS=interventricular septum, LVPW=left ventricular posterior wall, LVID=left ventricular internal diameter, FS=fractional shortening, EF=ejection fraction. Data are shown as mean ± SEM, n represents individual mice.

Table S2: Composition of the standard (SPD) and low protein (LPD) diet.

	SPD	LPD
**Energy (MJ/kg)**		
Gross energy	16.7	18.3
Metabolizable energy	13.6	15.6
**Crude Nutrients (%)**		
Dry matter	87.9	95.8
Crude protein	22.0	8.8
Crude fat	4.5	8.1
Crude fibre	3.9	5.0
Crude ash	6.8	5.5
N free extracts	50.8	68.4
Starch	34.0	56.1
Sugar	5.0	10.9

Table S3: Sequences of primers used for qRT-PCR experiments.

Gene	Forward Primer	Reverse Primer
Nppa	5´-CAGCATGGGCTCCTTCTCCAT-3´	5´-TGTACACAGGATTTGCTCCAATATG-3´
Nppb	5´-AGGACCAAGGCCTCACAAAA-3´	5´-TTGAGATATGTGTCACCTTGGAATTT-3´
Myh7	5´-CTAGAGTCAAAGTGGGCAACG-3´	5´-GTGTCACCATCCAGTTGAACA-3´
Col1a1	5´-CATGTTCAGCTTTGTGGACCT-3´	5´-ATCAAGCATACCTCGGGTTTC-3´
Col1a2	5´-TGTTGGCCCATCTGGTAAAGA-3´	5´-CAGGGAATCCGATGTTGCC-3´
Col3a1	5´-GGAACCTGGTTTCTTCTCACC-3´	5´-ATGTCATCGCAAAGGACAGAT-3´
Fn1	5´-GGCAGTGGTCATTTCAGATGCG-3´	5´-CTCCCTTTCCATTCCCGAGG-3´
Vim	5´-GTTTCCAAGCCTGACCTCACT-3´	5´-TGTCTCCGGTACTCGTTTGAC-3´
Tgfb1	5´-TGGAGCAACATGTGGAACTC-3´	5´-AGACAGCCACTCAGGCGTAT-3´
Tgfb3	5´-CTGGAAATCAGCATCCACTGT-3´	5´-CCATGGTCATCTTCATTGTCC-3´
Atf4	5´-CTATGGATGATGGCTTGGCC-3´	5´-GGTTTCCAGGTCATCCATTC-3´
Ddit3	5´-CTATATCTCATCCCCAGGAAAC-3´	5´-CATAGAACTCTGACTGGAATC-3´
Asns	5´-TCCAAGTATATTCGGAAGAAC-3´	5´-TCCAAGTATATTCGGAAGAAC-3´
Trib3	5´-GTCGCTTTGTCTTCAGCAAC-3´	5´-GTCGCTTTGTCTTCAGCAAC-3´
Vegfa	5´-GGACATCTTCCAGGAGTACC-3´	5´-GGCTTTGGTGAGGTTTGATCC-3´
Gapdh	5´-AGGTTGTCTCCTGCGACTTCA-3´	5´-CCAGGAAATGAGCTTGACAAAGTT-3´
